# Altered Patterns of Dynamic Functional Connectivity Underpin Reduced Expressions of Social–Emotional Reciprocity in Autistic Adults

**DOI:** 10.1002/aur.70010

**Published:** 2025-02-24

**Authors:** Kristína Czekóová, Radek Mareček, Rostislav Staněk, Calum Hartley, Klaus Kessler, Pavlína Hlavatá, Hana Ošlejšková, Milan Brázdil, Daniel Joel Shaw

**Affiliations:** ^1^ Behavioural and Social Neuroscience Research Group, Central European Institute of Technology (CEITEC) Masaryk University Brno Czechia; ^2^ Institute of Psychology Czech Academy of Sciences Brno Czechia; ^3^ First Department of Neurology, Faculty of Medicine Masaryk University Brno Czechia; ^4^ Multimodal and Functional Neuroimaging Laboratory (MAFIL), Central European Institute of Technology (CEITEC) Masaryk University Brno Czechia; ^5^ Department of Economics, Faculty of Economics and Administration Masaryk University Brno Czechia; ^6^ Department of Psychology Lancaster University Lancaster UK; ^7^ School of Psychology University College Dublin Dublin Ireland; ^8^ Department of Child Neurology University Hospital Brno and Masaryk University Brno Czechia; ^9^ First Department of Neurology St. Anne's University Hospital and Faculty of Medicine, Masaryk University Brno Czechia; ^10^ Department of Psychology, School of Life and Health Sciences Aston University Birmingham UK

**Keywords:** autism, dynamic functional connectivity, reciprocity, social interaction

## Abstract

To identify the neurocognitive mechanisms underpinning the social difficulties that characterize autism, we performed functional magnetic resonance imaging on pairs of autistic and non‐autistic adults simultaneously whilst they interacted with one another on the iterated Ultimatum Game (iUG)—an interactive task that emulates the reciprocal characteristic of naturalistic interpersonal exchanges. Two age‐matched sets of male–male dyads were investigated: 16 comprised an autistic Responder and a non‐autistic Proposer, and 19 comprised non‐autistic pairs of Responder and Proposer. Players' round‐by‐round behavior on the iUG was modeled as reciprocal choices, and dynamic functional connectivity (dFC) was measured to identify the neural mechanisms underpinning reciprocal behaviors. Behavioral expressions of reciprocity were significantly reduced in autistic compared with non‐autistic Responders, yet no such differences were observed between the non‐autistic Proposers in either set of dyads. Furthermore, we identified latent dFC states with temporal properties associated with reciprocity. Autistic interactants spent less time in brain states characterized by dynamic inter‐network integration and segregation among the Default Mode Network and cognitive control networks, suggesting that their reduced expressions of social–emotional reciprocity reflect less efficient reconfigurations among brain networks supporting flexible cognition and behavior. These findings advance our mechanistic understanding of the social difficulties characterizing autism.

**Summary**

- Autism is characterized largely by atypical expressions of social–emotional reciprocity, such as reduced sharing of interests and emotions during social interaction, but research has not yet identified the reasons for such behavior.
- To advance this field of research, we measured brain activity and behaviors of autistic and non‐autistic participants while they interacted with each other on an experimental task designed to emulate the reciprocal characteristic of real‐world social interaction.
- We found that autistic participants reciprocated their partner's behavior less than their non‐autistic counterparts, and this was associated with altered patterns of communication and integration among certain brain networks.
- Assuming that these reduced expressions of reciprocity shown by autistic participants provide an experimental index of their behavior during real‐world social interactions, this study identifies a potential mechanism behind the social difficulties reported by autistic people.

## 1 Introduction

Autism is a developmental condition characterized by difficulties in social interaction and communication (American Psychiatric Association 2013). Given the well‐established importance of meaningful interpersonal relationships for mental health (Santamaría‐García et al. 2020), it is essential that we identify the neurocognitive mechanisms giving rise to such social difficulties. While considerable progress has been made in this endeavor (Guo et al. 2024; Lord et al. 2020; Velikonja et al. 2019), our understanding remains limited because the experimental paradigms used most commonly fail to capture the conditions of real‐world social interaction under which interpersonal difficulties manifest—namely, the reciprocal dynamic through which social interactions evolve (Davis and Crompton 2021; Thaler et al. 2024). Consequently, studies often fail to detect atypical interpersonal behavior in autistic participants (Gernsbacher and Yergeau 2019) despite the social difficulties they report (Bylemans et al. 2023). Advancing our understanding of the neurocognitive systems that drive such difficulties in autism, therefore, requires us to capture them in real time during more naturalistic social encounters (Schilbach 2016; Thaler et al. 2024; Wheatley et al. 2019). To achieve this, the present study performed functional brain imaging on pairs of autistic and non‐autistic adults while they interacted with one another on a task designed to emulate the reciprocal characteristic of social interaction.

The difficulties in social interaction characterizing autism manifest predominantly as atypical social–emotional reciprocity, such as failures in back‐and‐forth conversation or the mutual sharing of interests and emotions (American Psychiatric Association 2013). Identifying the neurocognitive mechanisms underpinning these behavioral patterns therefore necessitates experimental paradigms that allow for variable expressions of interpersonal reciprocity. Reciprocity is defined as mutually dependent and symmetrical exchanges between individuals, and so its atypical expression in autism necessarily reflects the behavior of the non‐autistic individuals with whom autistic people interact most frequently (Gernsbacher 2006). The iterated version of the Ultimatum Game (iUG; Avrahami et al. 2013) is an experimental paradigm that offers an interactive setting for investigating this interpersonal dynamic. In each exchange, one player (Proposer) is required to divide a sum of money between themselves and their co‐player (Responder), and the latter can choose to accept or reject the proposed division. If the Responder accepts the Proposer's offer, the amount is divided accordingly; but if the offer is rejected, neither player receives any money. Unlike the one‐shot UG that ends after a single exchange, the iterated version is played repeatedly between the same players and permits expressions of bidirectional reciprocity. In previous studies, we developed a model of bidirectional reciprocity to estimate the behaviors of Proposer and Responder dyads on each exchange of the iUG (Shaw et al. 2019, 2018). This revealed that some players' behavior (e.g., Responders' rejections or acceptances) was driven by a reaction to how they felt they had been treated previously; they perceived greater utility in increasing their partner's relative payoff if they felt they had been treated fairly in earlier exchanges, but chose to decrease the partner's payoff in favor of their own if they felt they had been treated unfairly (positive and negative reciprocity, respectively). In contrast, other players adopted an unwavering strategy; by proposing only divisions that benefited themselves maximally or accepting only those that they considered to be fair, they forced their partner into a compromise over fairness and ultimate payoff.

To the best of our knowledge, no studies have utilized the iUG to explore reciprocity in dyads of autistic and non‐autistic players. Previous studies employing the one‐shot UG report a higher acceptance of unfair offers in autistic compared with non‐autistic Responders, suggesting the former have a lower aversion to unfairness (Hartley and Fisher 2018; Molins et al. 2024; Tei et al. 2018; Wang et al. 2019). These tendencies may be more apparent in autistic children than adults; however, older autistic individuals appear to implement learned fairness norms more consistently than non‐autistic players (Jin et al. 2020). Furthermore, behaviors shown in the one‐shot version might not transfer to the iUG because Responders' motivations will be very different when they know that Proposers can reciprocate their responses in turn. This non‐affordance for bidirectional reciprocity in the one‐shot format might explain why some studies report no differences in the behavior of autistic and non‐autistic players (Klapwijk et al. 2017; Trovato 2019; Woodcock et al. 2020). In this study, we apply our model of bidirectional reciprocity to the behavior of autistic and non‐autistic Responders playing the iUG with non‐autistic Proposers to determine if they show systematic differences in their expression of reciprocity over recursive monetary exchanges.

As with real‐world social interactions, iterative exchanges across the iUG represent a unique two‐in‐one dynamic that unfolds non‐linearly and unpredictably—through mutual expressions of reciprocity, both interactants' behavior at any one moment is simultaneously a consequence of and antecedent to their partner's actions. To coordinate behavior in such contexts, the brains of both interactants must be capable of recruiting and switching flexibly between networks of neural systems so they can respond and adapt continuously to the rapidly changing demands imposed by their interaction partner. This is referred to as dynamic functional connectivity (dFC; Hutchison et al. 2013). As such, alterations in dFC might give rise to atypical expressions of social‐emotional reciprocity in autism. Indeed, studies have shown that autism is associated with altered dFC when the brain is at rest (Roy and Uddin 2021), during which atypical patterns of integration and/or segregation are observed among several intrinsic brain networks—stable large‐scale neural circuits that transiently link distributed brain regions (Uddin et al. 2019). Frequent examples include the fronto‐parietal network, the ventral and dorsal attention networks, and the default mode network (Yeshurun et al. 2021). At rest, the brains of autistic people spend more time in states of hyper‐ and hypo‐connectivity among these brain networks (Li et al. 2020; Mash et al. 2019), and transitions among these dFC patterns differ when compared to their non‐autistic counterparts (Pan et al. 2023; Watanabe and Rees 2017). Interestingly, the coordinated integration of these intrinsic brain networks appears to play a crucial role in supporting interpersonal behavior in non‐autistic individuals; meta‐analytic data reveal their combined involvement during social cognitive functions (e.g., inferring others' intentions; Feng et al. 2021; Schurz et al. 2020), and our own research has shown that they integrate systematically during different types of interpersonal exchange (Shaw et al. 2023).

While these findings suggest that altered dFC might indeed underpin atypical expressions of social‐emotion reciprocity in autism, neuroimaging data acquired from this population during interpersonal settings remain scarce (Jasmin et al. 2023; Peng et al. 2024; Quiñones‐Camacho et al. 2021). This is an important limitation of existing research given that reciprocal behaviors and associated dFC patterns are necessarily interpersonal phenomena—they reflect both a reaction and precursor to a fellow interactant's behavior. In the present study, we estimated dFC and behavioral expressions of reciprocity from pairs of Proposers and Responders simultaneously while they interacted with one another on the iUG. This allowed us to compare behaviors measured with our model of bidirectional reciprocity and associated patterns of dFC between autistic and non‐autistic Responders, and between the non‐autistic Proposers with whom they interacted, while the pair co‐created a unique interpersonal context through bidirectional reciprocity.

The vast majority of existing studies into dFC in autism have utilized the sliding‐window technique (de Lacy et al. 2017; Hyatt et al. 2022; Mash et al. 2019; Rabany et al. 2019; Zhuang et al. 2023; Li et al. 2020)—a window of fixed length is progressed along a functional time series, and window‐by‐window changes in patterns of functional connectivity (FC) are calculated. The size of each window should be large enough to permit robust FC estimation at lower frequencies in that period yet small enough to detect potentially interesting between‐window transients. Window sizes around 30–60 s have been shown to achieve robust results in conventional acquisitions (for a review see Hutchison et al. 2013). However, this approach might miss the high‐frequency changes in FC that are likely to coordinate reciprocal behaviors during social interaction. An alternative approach is offered by state‐space modeling, whereby observed patterns of whole‐brain FC are represented as a function of independent and constantly changing latent brain states. Rather than estimating patterns of FC from data aggregated over discrete windows of predefined length, this data‐driven method applies matrix factorization to the entire time series to identify a set of latent space variables from which the observed data can be reconstructed and then estimates the posterior probability of each latent state at every time point (Taghia et al. 2018). With greater sensitivity to fleeting patterns of dFC, and by estimating each state's continuous evolution over time, state‐based approaches are more suited for uncovering the neurocognitive mechanisms associated with expressions of reciprocity during social interactions.

The aim of this study was to perform the first direct investigation of the neurocognitive mechanisms underpinning atypical social–emotional reciprocity in autistic adults during naturalistic social interactions. This advances the growing literature on alterations in functional brain connectivity in autism, which has focused almost exclusively on measuring brain activity in children at rest with analytical methods that do not capture the dynamism of latent whole‐brain states. To achieve this, we measured expressions of reciprocal behavior and concurrent patterns of dFC captured with a state‐space model during the iUG and compared them between autistic and non‐autistic Responders, and between the non‐autistic Proposers who interacted with these autistic or non‐autistic Responders in the game. This second‐person paradigm allowed us to capture the continuous evolution of latent dFC patterns associated with variable expressions of reciprocity as they occur during a given social interaction, which cannot be reproduced by scanning the brains of each interactant sequentially (Misaki et al. 2021; Redcay and Schilbach 2019; Shamay‐Tsoory and Mendelsohn 2019).

## 2 Methods

### 2.1 Participants

The dataset included two independent samples: one recruited specifically for this study that comprised pairs of autistic and non‐autistic adults (AA/NA dyads), and a second recruited for an earlier study (Shaw et al. 2018) that involved only non‐autistic adults (NA/NA dyads). Both samples were comprised only of males because sex differences in social interaction (Eagly and Wood 1991) and brain organization (Ingalhalikar et al. 2014) have the potential to confound measures of reciprocity and/or dFC during mixed‐sex exchanges.

#### 2.1.1 AA/NA Dyads

Seventeen autistic male adults (AA) diagnosed with Autism Spectrum Disorder (IQ > 80; *M* = 107, SE = 3; range 83–130) but no history of substance disorder or epilepsy were recruited from a database of former patients at University Hospital Brno. The other 17 were non‐autistic male adults (NA) recruited from the associates of Masaryk University (MU) and included individuals with no history of neurological or psychiatric diagnosis. These participants were paired into dyads matched on age and handedness (six left‐handed). To build on existing research that has focused on autistic Responders, AA participants always played the role of Responder and NA participants played the role of Proposer. The data from one dyad were unusable due to technical problems, leaving a final sample of 16 dyads (*M*_age_ = 24.50, ±5.96). The study was approved by the Research Ethics Committee of MU and the Ethics Committee of University Hospital Brno, and all participants provided written informed consent prior to the experimental procedure.

#### 2.1.2 NA/NA Dyads

This sample comprised 19 male–male dyads reported in (Shaw et al. 2018) that were age‐matched to the final AA/NA sample (*M*_age_ = 24.51, ±3.76). These participants were recruited from the associates of MU and reported no history of neurological or psychiatric diagnoses. This study was approved by the Research Ethics Committee of MU, and all participants provided informed consent prior to the experimental procedure.

### 2.2 Procedure

Both sets of dyads underwent a single testing session at the same research facility (CEITEC MU). The individuals comprising a dyad were introduced to one another for the first time on the day of the experiment and told explicitly that they would interact with the same individual to whom they had just been introduced. The experimental protocol for the AA/NA dyads comprised the Autism Spectrum Quotient and a single run of the iUG, and—for the AA sub‐group only—the Childhood Autism Rating Scale 2–HF. For the NA/NA dyads, the protocol comprised two successive runs of the iUG (each identical to the iUG played by the AA/NA dyads) followed by one run of the Dictator Game and two self‐report instruments measuring trait empathy (Interpersonal Reactivity Index; Davis 1983) and emotion regulation tendency (Action Control Scale; Kuhl 1994). Since data from the Dictator Game and responses to these questionnaires were not administered in the AA/NA sample, they are not analyzed in the present study. More importantly, in the analyses described henceforth, we only consider data from the first run of the iUG performed by NA/NA dyads to ensure comparable comparisons between the two dyad sets.

### 2.3 Materials

#### 2.3.1 The Iterated Ultimatum Game

**FIGURE 1:**
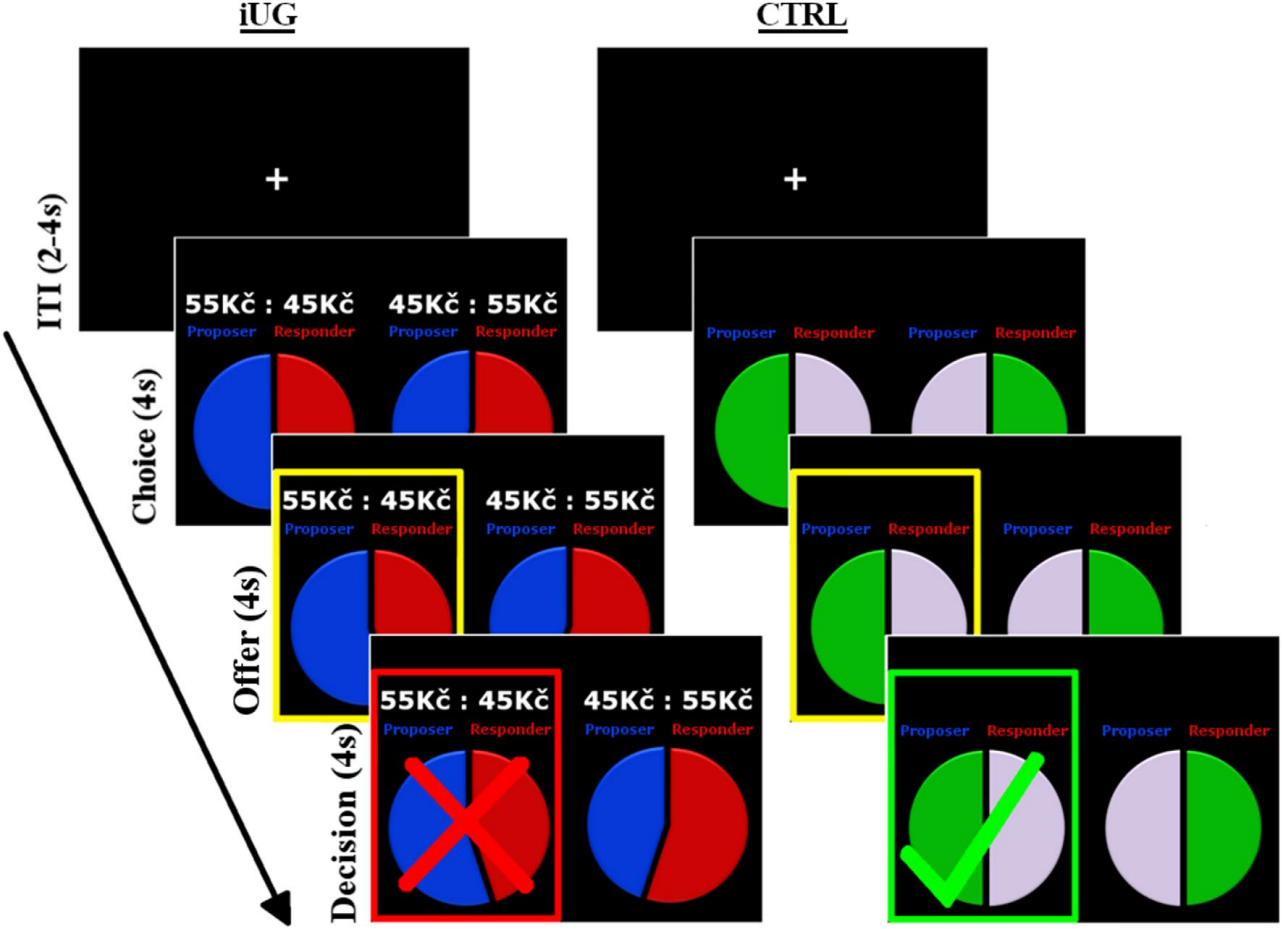
Experimental paradigm. On experimental (iUG) rounds, Proposers selected between two alternative monetary divisions to offer the Responder (Choice), after which their choice was presented, and Responders had to accept or reject the proposal (Offer). The Responder's decision was then presented subsequently (Decision). The rounds were separated by the jittered inter‐trial interval (ITI). The same sequence and timings were followed in Control (CTRL) rounds, but Proposers chose between two alternative divisions of color and the Responder decided whether to accept or reject the offer. In these examples, the unfair offer made by the Proposer on a Proposer–Responder (PR) round of the iUG is rejected by the Responder (left), while the offer made on the CTRL round is accepted (right).

The iUG was identical to the one employed in our earlier study (Shaw et al. 2018). All rounds consisted of three 4‐s periods (Choice, Offer, and Decision) and were separated by a jittered inter‐trial interval for 2–4 s. During the Choice period, players were presented with two alternative divisions of 100 CZK (the choice set; approximately €4) and Proposers were instructed to select one option to offer the Responder. Proposers could make their choice at any time during the 4‐s Choice period, but it was not indicated to the Responder until a subsequent Offer period. During the 4‐s Offer period, the Responder could either accept or reject the proposed division at any time, but their decision was not indicated to the Proposer until a subsequent 4‐s Decision period. To encourage reciprocity, choice sets always comprised two unequal divisions of money that differed in the direction of inequity: In Proposer–Responder (PR) rounds, Proposers were forced to make a choice between two divisions that presented the greater payout to either themselves or the Responder (e.g., 70:30 or 30:70). Conversely, in Proposer–Proposer (PP) rounds, both divisions were advantageous for the Proposer but varied in the magnitude of inequity (e.g., 70:30 or 60:40). For ease of interpretation, we refer to offers that maximize the Responder's payout as “fair” and those that maximize the Proposer's payout as “unfair”. As a means of localizing brain responses specific to these monetary exchanges, among them we intermixed 30 control (CTRL) rounds; following the same sequence, Proposers were instructed to choose between two different divisions of color, and Responders could accept or reject the proposed division (see Figure 1). The Supporting Information provides a full list of choice sets and the instructions given to participants.

The iUG included 30 PR, 30 PP, and 30 CTRL rounds presented in a pseudorandomised sequence optimized for contrast detection between conditions using a genetic algorithm (Wager and Nichols 2003). The number of remaining rounds was not disclosed to participants at any point. All the stimuli were presented to both players simultaneously throughout the entire interaction. At the end of the game, participants received the monetary outcome of three rounds selected randomly.

### 2.4 Autism Assessment

The Childhood Autism Rating Scale‐2HF (CARS‐2; Schopler et al. 2010) is a clinician‐rated scale for the assessment of autism symptomatology, consisting of 15 items, each rated from 1 (no abnormality) to 4 (severe abnormality; cut‐off = 28). The average CARS‐2 score in our sample was 30.5 (±2.9; range 27–37). The CARS‐2 was administered by an experienced psychologist who works routinely with autistic individuals (PH).

The Autism Spectrum Quotient (AQ; Baron‐Cohen et al. 2001) is a 50‐item questionnaire that measures autistic traits within different domains on a 4‐point Likert scale ranging from 1 (“Definitely agree”) to 4 (“Definitely disagree”). The assessed domains include social skills, attention switching, attention to detail, communication, and imagination. A score above 29 is considered indicative of autism (Broadbent et al. 2013). AQ scores differed significantly between autistic (*M* = 26.4, SD = 6.6) and non‐autistic individuals (*M* = 14.9, SD = 6.0; *t*_(30)_ = −5.19, *p* < 0.001).

### 2.5 MRI Data Acquisition and Preprocessing

Structural and functional brain images were acquired from both participants of a given pair simultaneously with one of two identical 3T Siemens Prisma scanners, and the images from both sets of dyads were pre‐processed identically using FMRIB's software library (FSL; Jenkinson et al. 2012). In addition to standard motion correction routines, we used Independent Component Analysis to identify artifactual signals arising from residual head motion and physiological noise and subsequently regressed these from the time series. Full details of the data acquisition and preprocessing protocols are provided in Supporting Information.

### 2.6 Reciprocity Modeling

To quantify each players' behavioral expression of reciprocity during the iUG, we adapted a Cox's reciprocity model (Cox et al. 2007). This model involves an evaluation of the choice set in terms of the final relative payout between the players, thereby accounting for inequity aversion (Bolton and Ockenfels 2000; Fehr and Schmidt 1999). Unlike other models (Molins et al. 2024), our adaptation incorporated data from both interacting players simultaneously to quantify the influence of emotional reactions to a co‐player's previous responses. This allowed us to capture more accurately the interdependency of dyadic interactions. Full specification of the estimation procedure is provided in the Supporting Information.

### 2.7 Dynamic Functional Connectivity

We extracted an average time series (690 measurements) from all voxels comprising each of the 400 non‐overlapping cortical parcels defined by Schaefer et al. (2018). This parcellation captures the topographical structure of the following seven functional networks detected reliably in resting‐state fMRI data (Thomas Yeo et al. 2011): the visual (VN) and somatomotor (SMN) networks, the dorsal and ventral attention networks (DAN and VAN), the limbic network (LN), the fronto‐parietal network (FPN), and the default mode network (DMN). Extracting time series from each network node enabled assessment of dynamic functional connectivity (dFC).

To these parcellated time‐series, we applied a state‐space dFC analysis. Unlike sliding‐window approaches, this technique assumes that observed brain activity at any moment is generated by a smaller number of underlying latent (hidden) states of brain connectivity with lower dimensionality. These latent states and their temporal evolution during the iUG were identified with Bayesian Mixture of Factor Analyzers (BMFA; Ghahramani and Beai 2000)—an alternative to Bayesian Switching Factor Analysis (Taghia et al. 2018) that does not model any temporal dependencies among states and is therefore influenced less by lower sampling frequencies (Ezaki et al. 2021). To identify role‐specific states of dFC expressed across both dyads and compare the temporal evolution of each state between them, BMFA was applied separately to the parcellated time‐series from all 35 Proposers and 35 Responders from the AA/NA and NA/NA dyads.

The number of states to extract was determined with Variational Bayesian Approximation: BMFA was computed for 2–15 States, and each number of states was estimated 40 times with random initializations and estimation convergence controlled by Free Energy and parameter change. Several metrics of fit were derived from this process: silhouette and point‐biserial correlation coefficients, and Davies–Bouldin, Calinski–Harabasz, and Dunn indices. The optimal number of brain states for each role was determined by the median of optimal values derived from each criterion (Brunet et al. 2011).

**FIGURE 2:**
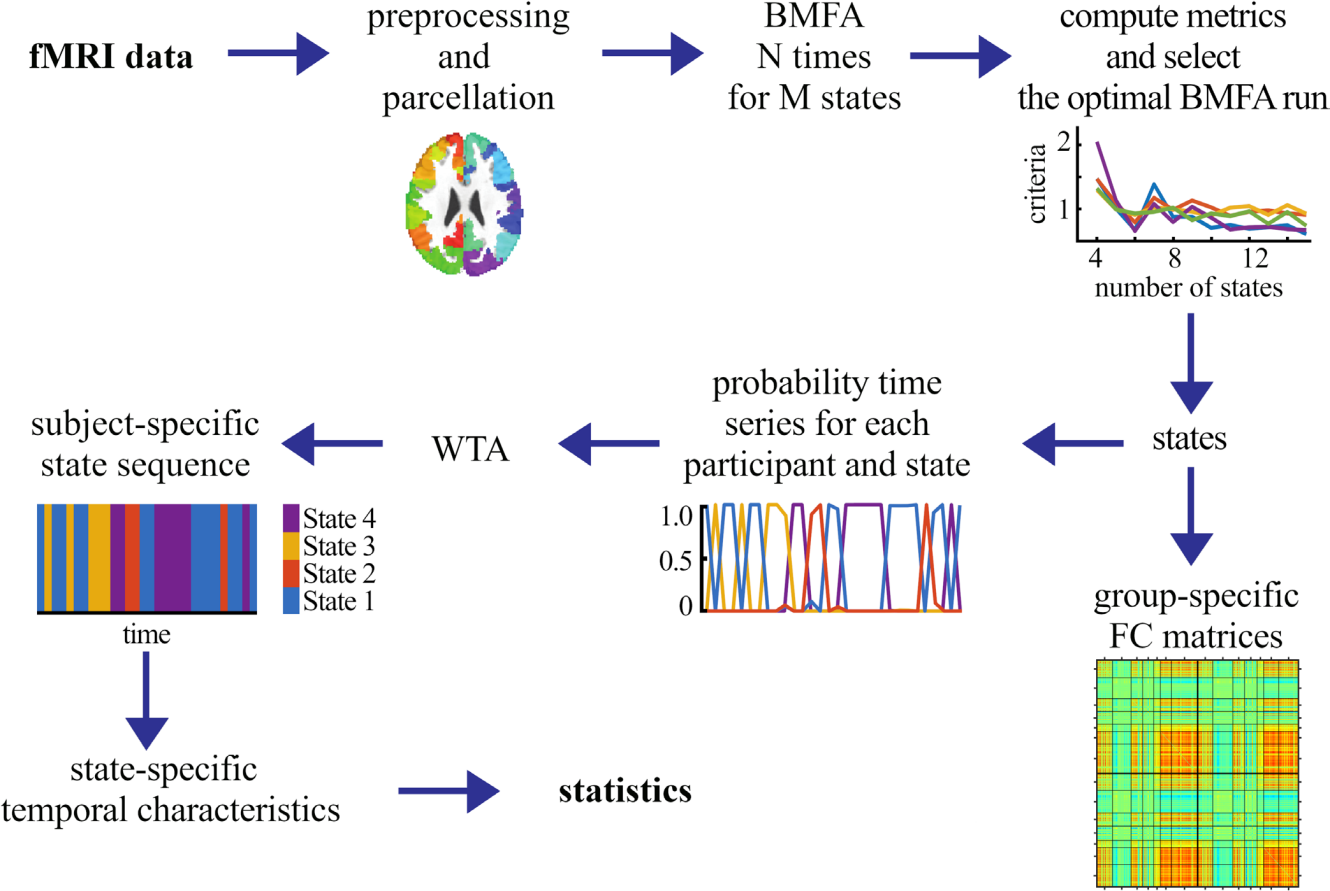
Processing pipeline for dFC. After pre‐processing, the fMRI data were parcellated into 400 regions. The representative time‐series for every region across all participants were entered into the Bayesian Mixture of Factor Analyzers (BMFA) model. The model was estimated repeatedly with random initialization and 2 to 15 States. The optimal run of the BMFA estimation was selected according to metacriterion comprising several metrics. Each resulting state was subsequently described by a group‐specific FC matrix and its probability time‐series for each participant. The winner‐takes‐all (WTA) approach was employed to construct participants' state sequences, which were used to compute temporal characteristics for each state. These characteristics were then subjected to statistical comparisons. The BMFA model was estimated separately for groups of Proposers and Responders.

For each of the optimal set of states, BMFA estimated its pattern of covariance among the 400 cortical parcels at the group level (across all 35 Proposers or Responders) and its posterior probability at every time point of each individual's full fMRI time‐series (throughout the entire iUG). The dominant state was identified at each time‐point—that is, the state with the highest probability of occurrence (winner‐takes‐all approach). The time‐series was then divided into epochs of a sustained dominant state, from which four temporal characteristics were calculated: coverage—the overall ratio of time frames in which that state dominates, occurrence—the number of times per minute that the state emerged dominant, lifetime—the mean duration of all epochs in which the state dominated, and transition probability—the probability with which each dominant state persisted from one moment to the next or transitioned to another state (within‐ or between‐state transitions). In addition to these task‐level temporal characteristics, we segmented and concatenated each individual's probability time series for a given state into rounds of each condition—PP, PR, and CTRL and subsequently calculated each state's coverage and transition probability across all rounds of a given condition, the latter computed only from consecutive time points. The analysis pipeline is illustrated in Figure 2.

### 2.8 Analytical Plan

To allow for comparisons with existing literature, iUG performance was measured with traditional composite indices (the frequency of fair/unfair offers and their acceptance/rejection) in addition to the reciprocity modeling. Using non‐parametric Mann–Whitney tests and False Discovery Rate to correct for multiple comparisons (Benjamini and Hochberg 2016), behavioral indices and temporal characteristics of role‐specific latent brain states were compared between the two groups of autistic and non‐autistic Responders, and between the two groups of non‐autistic Proposers from the AA/NA and NA/NA dyads. Sensitivity analysis performed in G*Power indicated that at *α* = 0.05, these between‐group comparisons could detect large effects (*r* ≥ 0.47) with 80% power (Faul et al. 2007). Brain‐behavior associations were investigated using the Spearman correlation coefficient.

## 3 Results

### 3.1 Behavior

Our adaptation of Cox's reciprocity model correctly estimated the responses of non‐autistic Proposers and autistic Responders comprising the AA/NA dyads on 72.1% (±12.6) and 77.9% (±18.7) of iUG exchanges, respectively; and the responses of non‐autistic Proposers and Responders from the NA/NA dyads on 71.6% (±8.6) and 81.9% (±10.7) of exchanges, respectively. Estimates of reciprocity from this modeling procedure were significantly lower among autistic Responders from the AA/NA dyads (*Mdn* = 0.04, IQR = 0.08) compared with their non‐autistic counterparts from NA/NA dyads (*Mdn* = 0.13, IQR = 0.04; *Z* = −3.21, *p* < 0.001, *r* = −0.54). More conventional iUG indices revealed no differences between autistic and non‐autistic Responders in their acceptance rates of unfair offers across PP (*Mdn*_AA/NA_ = 0.05, IQR = 0.90; *Mdn*_NA/NA_ = 0.50, IQR = 0.81; *Z* = −1.75, *p* = 0.082, *r* = −0.30) or PR rounds (*Mdn*_AA/NA_ = 0.47, IQR = 0.68; *Mdn*_NA/NA_ = 0.65, IQR = 0.32; *Z* =−1.29, *p* = 0.202, *r* = −0.22), but autistic Responders accepted fair offers significantly *less* frequently relative to their non‐autistic counterparts across both PP rounds (*Mdn*_AA/NA_ = 0.86, IQR = 0.30; *Mdn*_NA/NA_ = 1.00, IQR = 0.00; *Z* = −2.58, *p* = 0.009, *r* = −0.44) and PR rounds (*Mdn*_AA/NA_ = 0.88, IQR = 0.25; *Mdn*_NA/NA_ = 1.00, IQR = 0.00; *Z* = −3.59, *p* < 0.001, *r* = −0.61).

**FIGURE 3:**
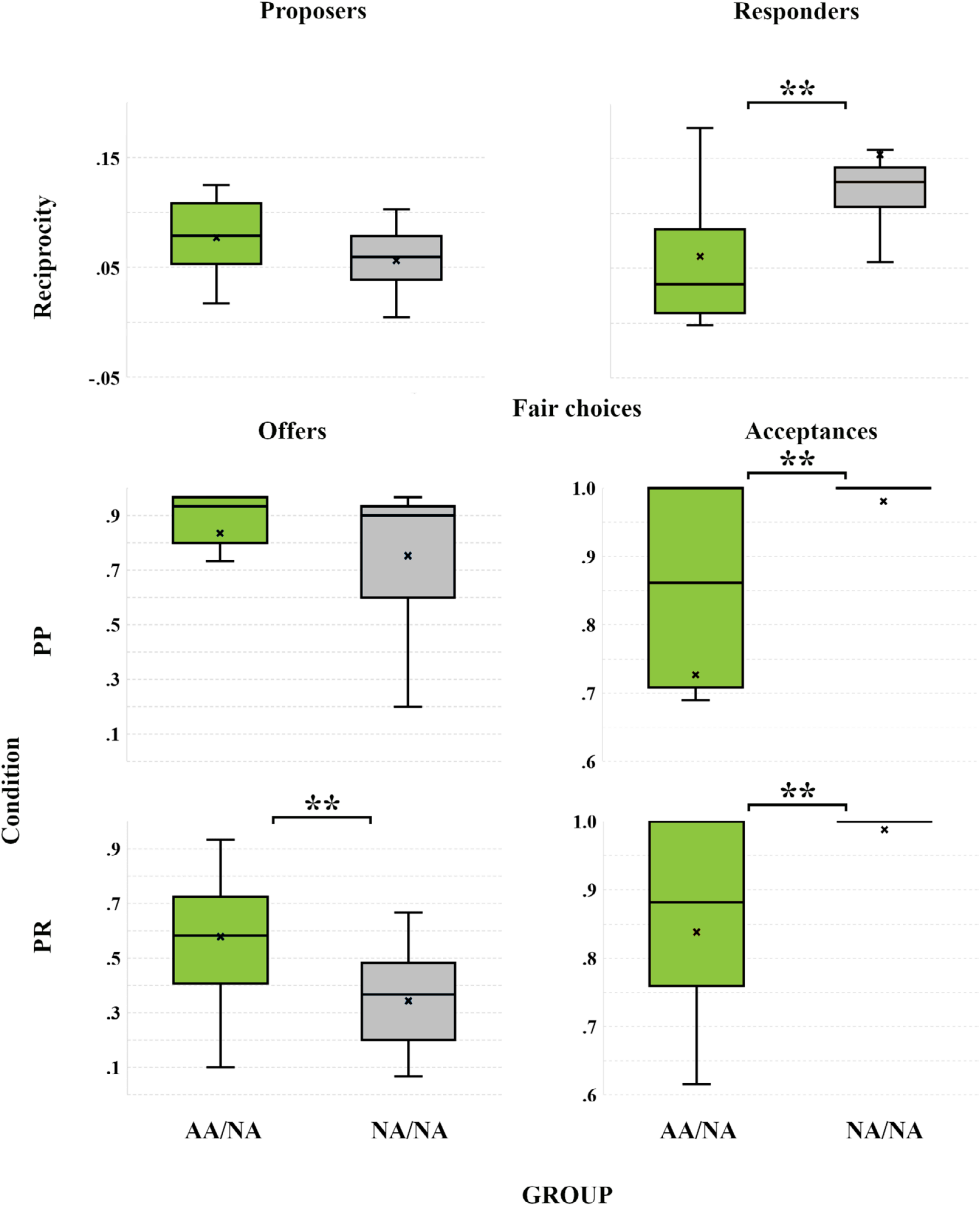
Comparisons of behavioral metrics between non‐autistic Proposers (left) comprising the AA/NA (green) or NA/NA dyads (gray), or between autistic and non‐autistic Responders (right) comprising AA/NA (green) or NA/NA dyads (gray). Top to bottom: Role‐specific reciprocity parameters calculated from trial‐by‐trial monetary exchanges of the iUG, and proportions of fair offers (those presenting the least advantageous inequity from the Proposer's perspective) and their acceptances across Proposer–Proposer (PP) and Proposer–Responder (PR) rounds. Boxplots illustrate medians (horizontal lines) within interquartile ranges, with means presented as crosses. Note the ceiling effects where non‐autistic Responders from NA/NA dyads accepted almost all the fair offers. *Note*: *p < 0.05, **p < 0.01.

In contrast, reciprocity estimates of non‐autistic Proposers from both sets of dyads were comparable (*Mdn*_AA/NA_ = 0.08, IQR = 0.06; *Mdn*_NA/NA_ = 0.06, IQR = 0.04; *Z* = −1.76, *p* = 0.082, *r* = −0.30), but the proportion of fair offers on PR rounds (favoring either Proposer or Responder) was significantly lower among non‐autistic Proposers who interacted with non‐autistic Responders (*Mdn*_NA/NA_ = 0.37, IQR = 0.30; *Mdn*_AA/NA_ = 0.58, IQR = 0.42; *Z* = −2.90, *p* = 0.003, *r* = −0.49). No such difference was observed on PP rounds, however, when the fairer offer represented a choice of less over more advantageous inequity from the Proposer's perspective (*Mdn*_AA/NA_ = 0.93, IQR = 0.17; *Mdn*_NA/NA_ = 0.90, IQR = 0.37; *Z* = −1.04, *p* = 0.306, *r* = −0.18). The results of these direct comparisons between the AA/NA and NA/NA dyads for Responders and Proposers are illustrated in Figure 3.

### 3.2 Dynamic Functional Brain Connectivity

#### 3.2.1 Latent Brain States

**FIGURE 4:**
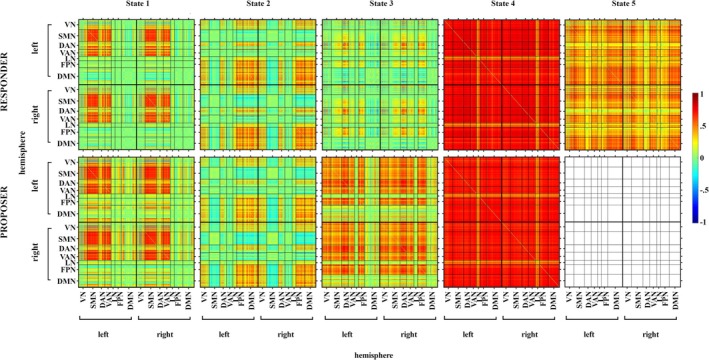
Latent brain states. Matrices depict functional connectivity among all nodes of the seven brain networks characterizing each of the latent brain states identified from Responders (top) and Proposers (bottom) across both AA/NA and NA/NA dyads. Functional connectivity is expressed as pairwise Pearson correlation coefficients computed from co‐variances identified with BMFA. Matrices are organized by brain networks in each hemisphere. DAN = dorsal attentional network, DMN = default mode network, FPN = fronto‐parietal network, LN = limbic network, SMN = somato‐motor network, VAN = ventral attentional network, VN = visual network.

The median metacriterion indicated that 4‐ and 5‐State solutions were optimal for Proposer and Responder roles, respectively (see Figure S1). As illustrated in Figure 4, three of four role‐specific latent dFC states showed striking similarity across both dyad sets, with all states exhibiting high interhemispheric symmetry.

State 1 is characterized by strong positive correlations between SMN and VAN, and positive but somewhat less consistent correlations among the DAN and VN. This state also comprises a mixture of weaker positive and negative correlations among selected nodes of the DMN, FPN, and LN networks.

State 2 represents positive correlations among the DMN, FPN, VN, and to some degree between the DAN and LN. Interactions between the attentional networks DAN and VAN are less strong in this state and consist of both positive and negative associations. Similarly, the SMN shows negative correlations with other networks, particularly the FPN, DMN, and certain nodes of VAN.

The pattern of State 3 differs markedly between the two roles: In Proposers, this state consists of relatively strong and consistent correlations between most of the networks except the DMN and LN, where both positive and negative associations emerged. In contrast, this state is connected more weakly in Responders—while the DAN and FPN are largely co‐activated, less comprehensive functional connectivity emerged within and between VN and VAN, and the DMN is largely segregated.

State 4 can be characterized as a whole‐brain hyper‐connected state. It is represented by very strong associations among all networks except the LN, which seems to be co‐engaged much less.

State 5 in Responders can be summarized as another densely (albeit less strongly) inter‐connected state capturing strong interactions among the investigated networks, with the exception of the DAN.

Patterns of hyperconnectivity such as those comprising State 4 might reflect global brain signal (GS), which has been attributed traditionally to measurement artifacts. We therefore conducted analyses to determine the extent to which the GS contributed to each of the latent brain states we have observed. For each participant, their GS was fitted to the time‐series extracted from each of the 400 parcels to determine the proportion of covariance explained by the GS. While the covariance pattern of State 4 was indeed explained by GS to the greatest degree in both Proposers and Responders, it contained a large amount of covariance unexplained by GS variability. Furthermore, GS contributed only marginally to the covariance of the other latent states. The results of these analyses are presented in Figures S2 and S3.

#### 3.2.2 Group Differences

**FIGURE 5:**
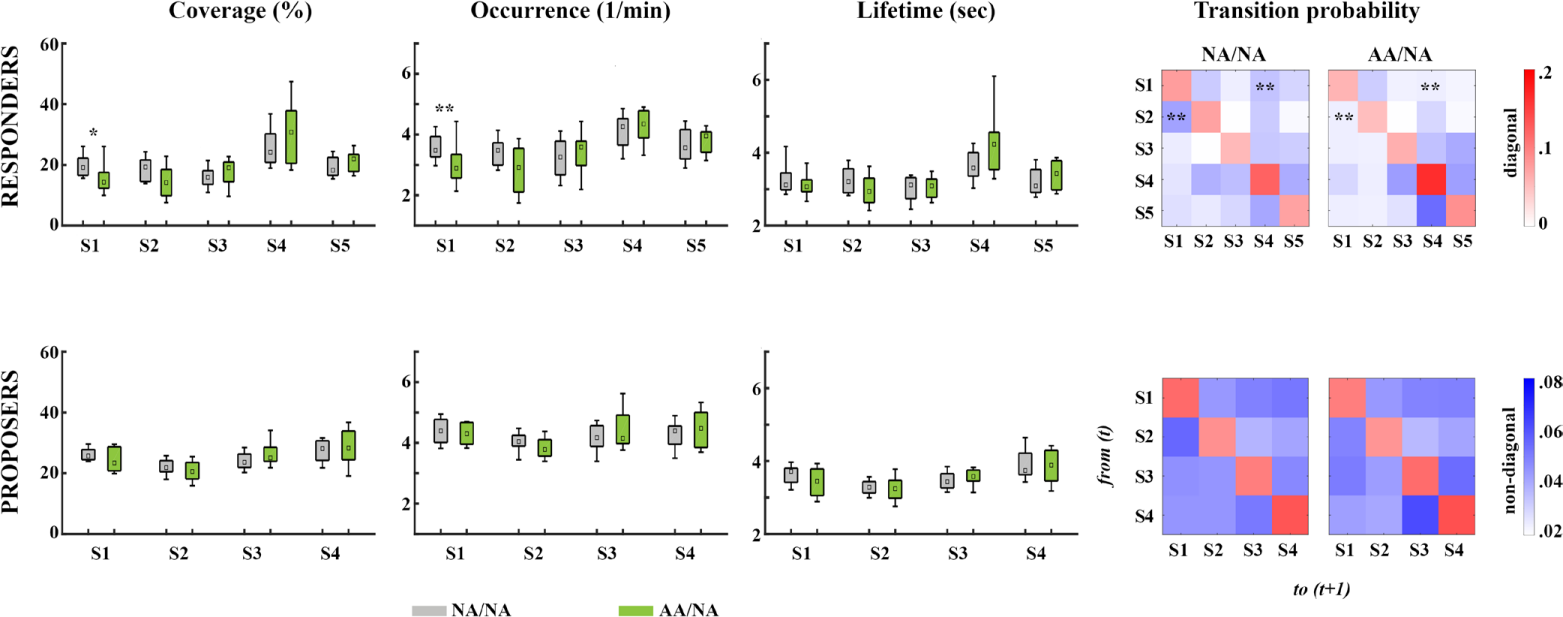
Differences between players of AA/NA (green) and NA/NA (gray) dyads in the temporal characteristics of latent brain states computed across the entire iUG: The bar charts present medians and interquartile ranges. S1–S5 = State 1–State 5; *p < 0.05; **p < 0.01.

Across the entire iUG, autistic Responders demonstrated significantly lower coverage (*Z* = −2.68, *p* = 0.007; *r* = −0.45) and occurrence (*Z* = −2.93, *p* = 0.003; *r* = −0.50) of State 1. Correspondingly, transitions from State 1 to State 4 (*Z* = −3.49, *p* < 0.001; *r* = −0.59) and from State 2 to State 1 (*Z* = −3.39, *p* < 0.001; *r* = −0.57) were significantly less probable in autistic compared with non‐autistic Responders. No other differences survived the FDR correction. In contrast, non‐autistic Proposers from the two sets of dyads did not differ in any of the indices (see Figure 5).

**FIGURE 6:**
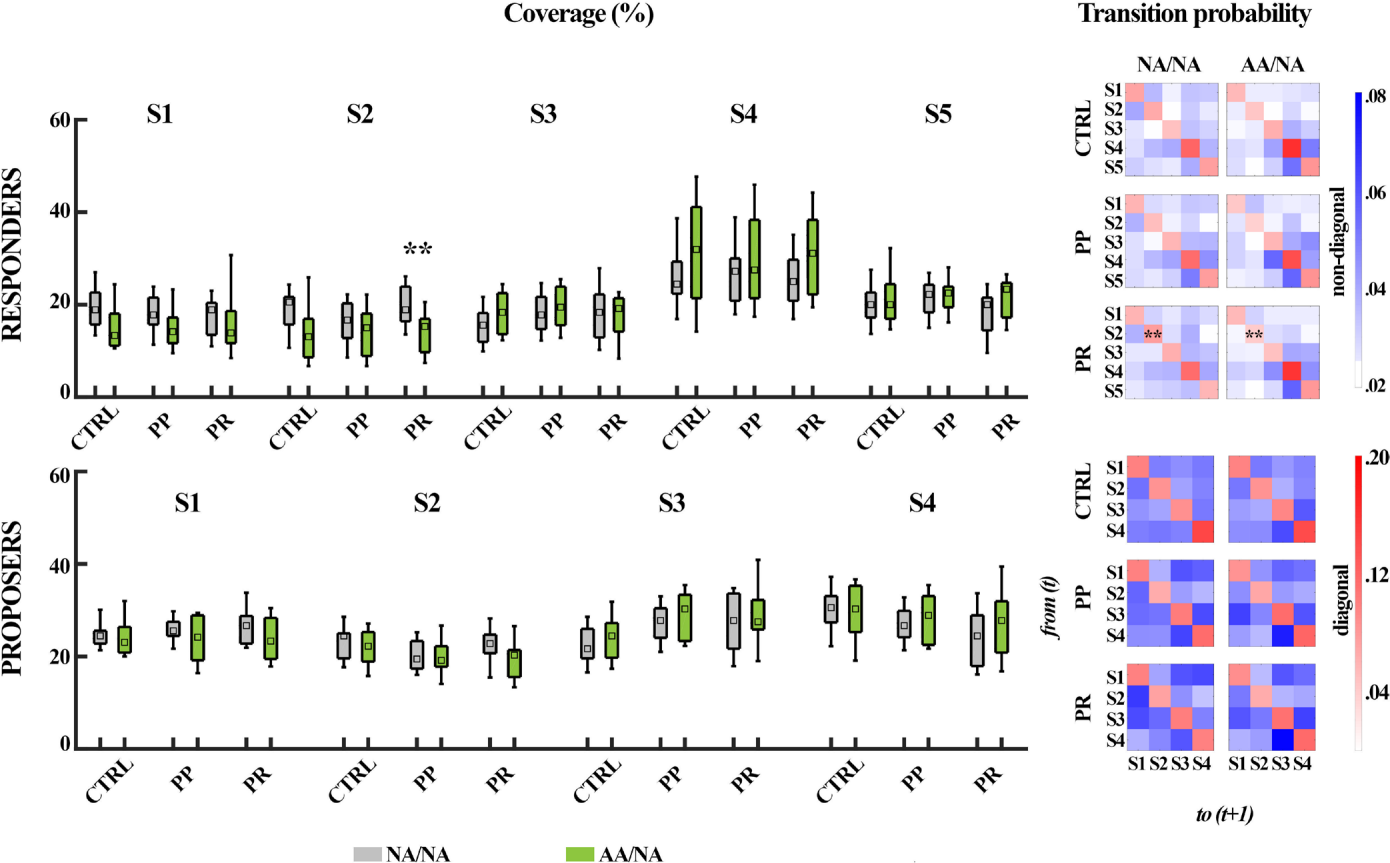
Condition‐specific differences in the temporal characteristics of latent brain states between players of NA/NA (gray) and AA/NA dyads (green). Boxplots present medians and interquartile ranges. CTRL = control, PP=Proposer–Proposer, PR = Proposer–Responder, S1–S5 = State 1–State 5; *p < 0.05; **p < 0.01.

When examining condition‐specific metrics, autistic Responders showed significantly less coverage than their non‐autistic counterparts for State 2 in the PR condition (*Z* = −2.73, *p* = 0.005; *r* = −0.46). Perhaps for this reason, there were significantly fewer within‐state transitions for State 2 in the PR condition for autistic compared with non‐autistic Responders (*Z* = −2.99, *p = 0*.002; *r* = −0.51; see Figure 6).

### 3.3 Brain‐Behavior Relationships

In non‐autistic Responders, player‐specific reciprocity parameters were correlated positively with the lifetime of State 2 (*ρ* = 0.539; *p* = 0.017; 95% CI [0.10, 0.80]) and negatively with State 1 to State 4 transitions (*ρ* = −0.660; *p* = 0.002; 95% CI [−0.86, −0.28]). In other words, stronger reciprocity in non‐autistic Responders was associated with a lower probability of transitioning from State 1 to State 4—a globally hyperconnected state with variability explained partly by the GS. In contrast, the only metrics related to reciprocity expressed by autistic Responders were the transition from the hyperconnected State 4 to State 2, the latter characterized by an integration of DMN, FPN, DAN, and VN and segregation of SMN and VAN (*ρ* = −0.534, *p* = 0.033; 95% CI [−0.82, −0.04])—the lower probability of transitioning between these latent states was associated with stronger reciprocity.

**FIGURE 7:**
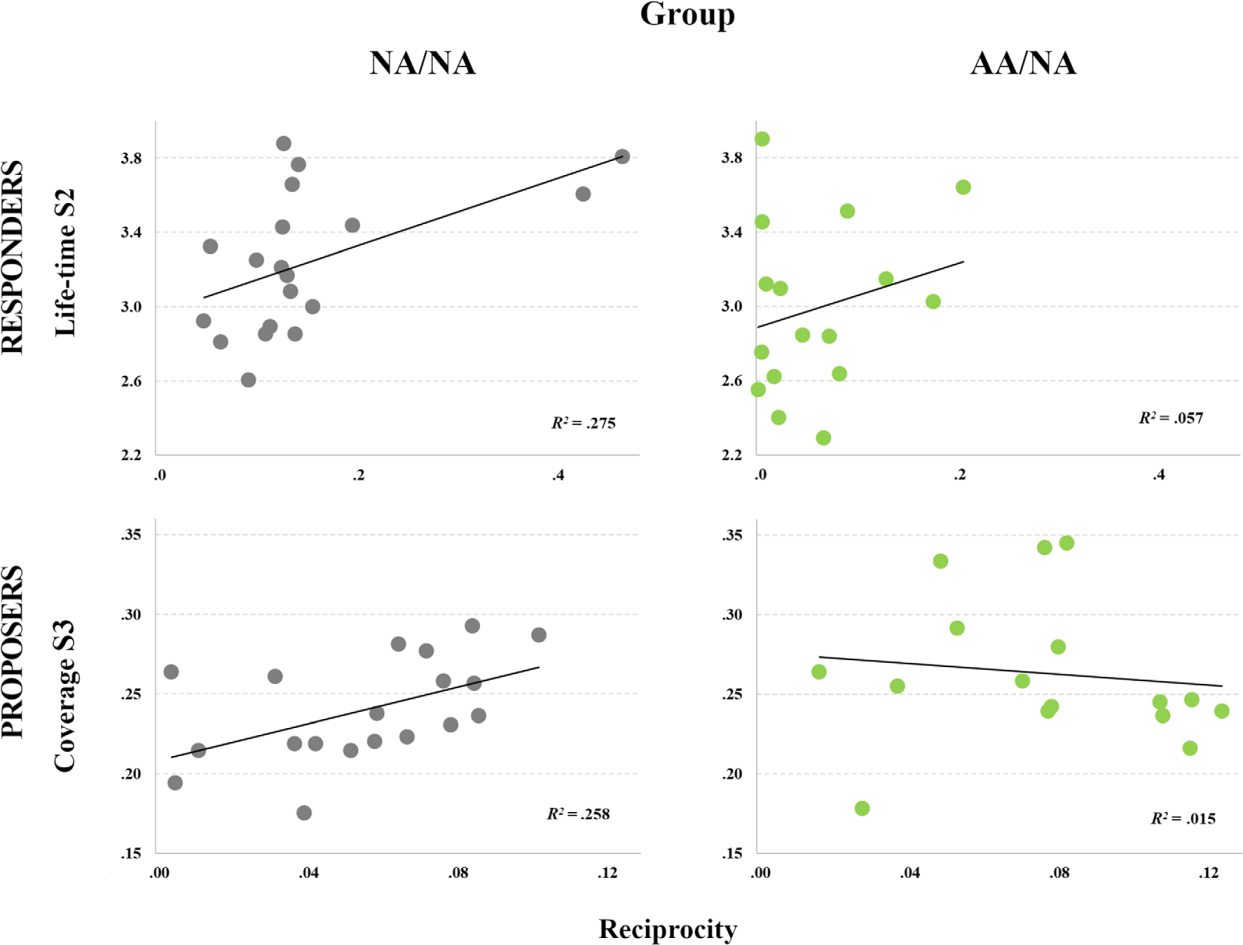
Brain‐behavior relationships. Associations between player‐specific reciprocity parameters estimated with the modeling procedure and the temporal characteristics of latent brain states exhibited by Responders (top) and Proposers (bottom). *Note*: NA/NA = dyads comprising a non‐autistic Responder and Proposer (gray), AA/NA = dyads comprising an autistic Responder and non‐autistic adult Proposer (green).

In Proposers from NA/NA dyads, a greater expression of reciprocal behavior was associated with a larger coverage (*ρ* = 0.521; *p* = 0.022; 95% CI [0.07, 0.79]) and a higher occurrence (*ρ* = 0.561; *p* = 0.012; 95% CI [0.13, 0.81]) of State 3, characterized by consistent and relatively strong integration of the SMN, VAN, DAN, FPN, and VN, and segregation of the LN and (less consistent) engagement of the DMN (see Figure 7). Tables S2–S5 present all brain‐behavior correlation matrices.

## 4 Discussion

This is the first investigation of dynamic functional connectivity (dFC) as it unfolds in the brains of autistic and non‐autistic adults while they engage with one another in naturalistic social exchanges. By applying a model of reciprocity to interactive behavior on the iterated Ultimatum Game (iUG), we show evidence of reduced expressions of reciprocity in autistic compared with non‐autistic adults. Furthermore, using a state‐space analysis of dFC, we reveal that these reduced expressions of reciprocity are associated with latent brain states defined by less dynamic integration and segregation among large‐scale brain networks—particularly among the DMN and cognitive control networks (e.g., FPN, VAN). Together, these findings offer novel mechanistic insights into the neurocognitive mechanisms that give rise to atypical expressions of social–emotional reciprocity characterizing autism.

The reduced expressions of reciprocity that we have observed converge with other reports of altered reciprocal behavior measured during interaction between autistic and non‐autistic adults: autistic individuals have been shown to make fewer reciprocal contributions and express lower reciprocal flexibility during (non‐verbal) interaction (Backer van Ommeren et al. 2022), exhibit less dynamic updating of communicative signals during an ongoing interaction (Wadge et al. 2019) and a greater reliance on prior information about a co‐player's reputation despite repeated contradictory experiences (Maurer et al. 2018). Our observation of similar rejection rates to unfair offers but fewer acceptances of fair offers (i.e., those favoring Responders) in autistic compared with non‐autistic Responders diverges from previous studies, however, which report more frequent acceptance of unfair offers and a comparable acceptance rate for fair offers in autistic relative to non‐autistic Responders during the one‐shot UG (Jin et al. 2020; Molins et al. 2024; Tei et al. 2018; Wang et al. 2019; but see Sally and Hill 2006; Trovato 2019; Woodcock et al. 2020). These discrepancies likely reflect a fundamental difference between the two paradigms—namely, the influence of Responders' decisions on subsequent Proposer offers during iterated exchanges. Nevertheless, similar interpretations can be applied to these findings: they might reflect differences between autistic and non‐autistic Responders in their ability, willingness, or strategic motivation to infer the intentions behind Proposers' offers (Sally and Hill 2006). This would have been exacerbated in the current study by the fact that autistic individuals were never given the chance to take the role of Proposer and thus adopt their alternative perspective. Alternatively, autistic Responders may have implemented more consistent and/or objective fairness norms when responding to contextual changes (Forbes et al. 2023; Forgeot D'Arc et al. 2020).

The tendency for autistic individuals to respond with greater consistency to contextual manipulations has been documented across various economic paradigms. Examples include inflexibility to changes in the social closeness between interactants or the explicit motivations of a supposed co‐player (Forbes et al. 2023; Forgeot D'Arc et al. 2020), the presence or absence of punishment (Hase et al. 2023), or outcome framing effects (Molins et al. 2024). These findings suggest that autistic Responders on the iUG might have behaved according to objective fairness norms rather than interpreting Proposers' offers as (communicative) signals of their intentions (e.g., Proposers offering divisions that maximally disadvantage them as a means of communicating their cooperative intention). This would be consistent with the general strategy applied by autistic individuals on a gambling task irrespective of the intentions of a supposed co‐player (Forgeot D'Arc et al. 2020). In turn, non‐autistic Proposers might not have understood the motivation of autistic Responders to reject fair offers and attempted to increase the number of acceptances by offering monetary divisions that disadvantaged themselves; such offers were significantly more frequent in AA/NA compared with NA/NA dyads. This could reflect a mismatch between autistic and non‐autistic (social) cognitive processing styles, as suggested by the double empathy problem (Milton 2012). Alternatively, autistic Responders might have found it more difficult to adjust their decisions flexibly during the event‐related design we employed in the present study, whereby the nature of choice sets changed on each successive round. The decision‐making of autistic adults in ambiguous situations has been shown to be affected by the predictability of the outcome (Macchia et al. 2024), and so the uncertainty of iUG interactions could have played a role in their less flexible (reciprocal) behavior. In this light, altered social–emotional reciprocity in autism might reflect a reduced sensitivity to contextual changes (cognitive inflexibility) in the face of rapidly and unpredictably changing demands encountered during real‐time social exchanges (Forgeot D'Arc et al. 2020; Tei et al. 2018).

Our interpretation that reduced reciprocity in autistic Responders reflects a more general decrease in sensitivity to context aligns with the state‐space dFC patterns we have revealed. The differences we have identified in the dynamics of latent brain states between autistic and non‐autistic Responders are largely consistent with existing evidence of altered FC in the autistic brain at rest: less frequent brain‐state transitions (de Lacy et al. 2017; Watanabe and Rees 2017), atypical inter‐ and intra‐network transitions (hypo‐ and hyper‐connectivity; Pan et al. 2023), atypical within‐network nodal relationships (Yue et al. 2022) and fewer dissociable states of time‐varying connectivity among brain networks (Rabany et al. 2019). Such aberrant connectivity is reported most frequently among the DMN, V/DAN, and the FPN, but also within VN and SMN, which are interpreted to reflect more stable neural processing (Ilioska et al. 2023; Watanabe and Rees 2017; Wang et al. 2022). Our data showed that autistic and non‐autistic Responders differ specifically in the dynamics of latent brain states that involve consistent and complex interplay (coordinated integration and segregation) of the same large‐scale networks (States 1 and 2). More specifically, these two latent states differed in the degree of connectivity between the DMN and the set of networks implicated in cognitive control (i.e., FPN, DAN). This finding confirms the importance of these network configurations in social cognitive processing (Maliske and Kanske 2022) and is in line with reduced within‐ and between‐DMN connectivity shown in autistic individuals elsewhere (de Lacy et al. 2017; Pan et al. 2023; Watanabe and Rees 2017). Differences in State 2 dynamics were specific to the PR condition, where the player's decision involves the strongest conflict between self and other interests, and so the integration of the DMN could be indicative of more effortful and controlled cognitive processing in response to increasing task complexity, during which the DMN is believed to assist in the formation of abstract representations by integrating information from other brain networks (Yeshurun et al. 2021). The positive relationship between State 2 and expressions of reciprocity further suggests that the duration of coordinated activity between the DMN and FPN and the concurrent disengagement of SMN and VAN are important for interactive behaviors in non‐autistic Responders. Similar patterns of strong functional connections between the same networks were characteristic of latent brain states identified in our earlier work, where they differentiated between cooperative and competitive exchanges in non‐autistic interactants (Shaw et al. 2023).

On the other hand, a negative covariance between the DMN and cognitive control networks characterizing State 1 should emerge when there is a need for external focus on social signals that require immediate response (Schurz et al. 2020). Social interactions necessitate coordinated internally and externally focused mentation, requiring a flexible balance between network configuration patterns (Maliske and Kanske 2022); to effectively reciprocate a partner's behavior, we must carefully monitor their behaviors (e.g., a Proposer's pattern of offers) in order to generate inferences about their momentary motivational, intentional, and affective state, and adjust our own behavior accordingly. The lower coverage of State 1 in autistic Responders might reflect less efficient network reconfigurations supporting these cognitive processes, which could reduce social–emotional reciprocity and make social encounters more challenging. However, similar direct comparisons between autistic and non‐autistic samples might not be appropriate. While higher general cognitive abilities have been found to be associated with greater stability of brain dynamics at rest in autistic adults, they are correlated with more flexible brain dynamics in non‐autistic adults (Watanabe and Rees 2017). These differences in relationships between brain dynamics and cognition highlight the need for further investigations.

State 4 was prominent in both Proposers and Responders during the iUG. This hyperconnected state has been observed in earlier studies, including those that have employed a sliding‐window approach (e.g., de Lacy et al. 2017; Mash et al. 2019). In the present study, we show that a substantial amount of the covariance captured by this latent state was shared by the global signal, which is interpreted commonly to reflect artifactual signals (e.g., respiration; Zhang and Northoff 2022). Recent research suggests that the global signal may, in fact, contain important information; however, linking it to arousal, task performance (Zhang et al. 2020), and differences between clinical and non‐clinical groups (Gotts et al. 2013). These findings have been taken as evidence that global brain activity reflects an equilibrium of internal signals and task‐related demands, and its alteration in clinical groups may underpin differences in cognitive processing (Zhang and Northoff 2022). State 4 may therefore reflect (at least in part) this global internal‐to‐external coordination. Reduced transition probabilities to (in NA Responders) and from (AA Responders) State 4 were associated with stronger expressions of reciprocity, potentially indexing a form of dysregulation or internal‐external disequilibrium. This finding should encourage future research on autism and social interaction more generally to consider the role of global brain activity. Interestingly, task‐related hyperconnectivity that seemed to facilitate typical conversation has been recently reported during a naturalistic interactive setting in autistic adults even after accounting for global activity (Jasmin et al. 2019, 2023).

It is important to acknowledge that some limitations of the present study can be overcome in future research. Although our sample sizes were sufficient to detect the large between‐group differences we have revealed, these findings require replication in larger and more heterogeneous autistic samples. Further, we compared dyads comprising autistic Responders and non‐autistic Proposers with those comprised of non‐autistic participants. While social interactions between autistic and non‐autistic individuals are most common in everyday life, it has been shown repeatedly that social exchanges between two autistic individuals can be equally efficient and achieve comparable levels of rapport as those observed in non‐autistic dyads (Crompton et al. 2020; Rifai et al. 2022). Similarly, given known sex differences in social cognition (Proverbio 2023) and brain connectivity in the autistic population (Roy and Uddin 2021), our focus on male–male dyads precludes any generalization of our findings to autistic females. Second, although a lack of framing effects suggests that emotional responses do not contribute significantly to the performance of autistic adults on (non‐interactive) economic games (Molins et al. 2024), emotional management could influence fairness processing and reciprocity in autistic individuals (Jin et al. 2020). Indeed, our reciprocity model incorporated a given player's emotional reaction to the prior behavior of their co‐player. Unfortunately, behavioral measures of emotion regulation were not included in this study and we encourage future studies to examine the role of emotion regulation abilities in reciprocity expression (Woodcock et al. 2020). Subjective perceptions of interaction quality (Rifai et al. 2022) from both dyad members should also be included to complement more objective behavioral indices of reciprocity. Next, we examined dFC among brain networks defined by a cortical parcellation derived from the brains of non‐autistic samples (Schaefer et al. 2018). However, studies have reported increased variability in the topographical organization of brain networks in the autistic population, with the DMN, DAN, SMN, and VAN shown to be shifted from their typical locations (Benkarim et al. 2021; Nunes et al. 2019) and such variability could have contributed to our results. Finally, our current design precluded investigation of between‐brain coupling in dFC patterns; players' choices were presented to their co‐players after a variable delay, and so there was no fixed contingency between the actions of one player and the brain responses of another. By showing players the choices of their partner in real time, future studies can start to identify if the neural alignment that we have observed previously in discrete brain regions (Shaw et al. 2018) extends to whole‐brain patterns of dFC.

By acquiring behavioral and fMRI data from pairs of autistic and non‐autistic males engaged in naturalistic bidirectional social exchanges and applying sophisticated data modeling techniques to these data, this study reveals for the first time that reduced expressions of interpersonal reciprocity in autism are associated with altered temporal characteristics of latent brain states characterized by coordinated inter‐network integration and segregation of cognitive networks and DMN. Assuming that the reduced expressions of reciprocity shown by autistic Responders provide an experimental index of their real‐world social interaction, this study captured socially disadvantageous behaviors that can have negative consequences for relationship building.

These results highlight the importance of interactive paradigms, dual‐brain imaging, and whole‐brain dFC analyses in autism research, especially in adult populations. First, the study advances our understanding of the neural underpinnings of social difficulties reported by autistic individuals. Our findings suggest the potential benefit of investigating the role of global brain activity in autistic adults, particularly during social interactions or more naturalistic social contexts, which could help to reconcile the hypo‐ versus hyper‐connectivity debate (Mash et al. 2019). Second, our interactive task could be employed as an objective measure of social–emotional reciprocity in intervention evaluation. Third, our findings suggest that autistic people use consistent/less reciprocal behavioral strategies during their social interactions with non‐autistic people. Understanding these differences in cognitive styles could facilitate communication between non‐autistic and autistic individuals. Using interactive experimental paradigms and dual‐brain imaging, future research should investigate if and how these same behavioral strategies and associated neural processes change in autistic dyads or with people with whom autistic individuals have more social proximity (e.g., friends, family).

## Conflicts of Interest

The authors declare no conflicts of interest.

## Supporting information


**Data S1.** Supporting Information.

## Data Availability

All experimental materials, protocol, and analysis codes are available publicly at https://osf.io/z7v5k/. Behavioral and brain imaging data are available upon reasonable request to the corresponding author, following approval for data sharing from the Research Ethics Committee of Masaryk University.

## References

[aur70010-bib-0001] American Psychiatric Association . 2013. “Diagnostic and Statistical Manual of Mental Disorders.”

[aur70010-bib-0002] Avrahami, J. , W. Güth , R. Hertwig , Y. Kareev , and H. Otsubo . 2013. “Learning (Not) to Yield: An Experimental Study of Evolving Ultimatum Game Behavior.” Journal of Socio‐Economics 47: 47–54. 10.1016/j.socec.2013.08.009.

[aur70010-bib-0003] Baron‐Cohen, S. , S. Wheelwright , R. Skinner , J. Martin , and E. Clubley . 2001. “The Autism‐Spectrum Quotient (AQ): Evidence From Asperger Syndrome/High‐Functioning Autism, Males and Females, Scientists and Mathematicians.” Journal of Autism and Developmental Disorders 31, no. 1: 5–17. 10.1023/A:1005653411471. 11439754

[aur70010-bib-0004] Benjamini, Y. , and Y. Hochberg . 2016. “Controlling the False Discovery Rate: A Practical and Powerful Approach to Multiple Testing.” Journal of the Royal Statistical Society, Series B 57, no. 1: 289–300.

[aur70010-bib-0005] Benkarim, O. , C. Paquola , B. y. Park , et al. 2021. “Connectivity Alterations in Autism Reflect Functional Idiosyncrasy.” Communications Biology 4, no. 1: 1078. 10.1038/s42003-021-02572-6. 34526654 PMC8443598

[aur70010-bib-0006] Bolton, B. G. E. , and A. Ockenfels . 2000. “A Theory of Equity, Reciprocity, and Competition.” American Economic Review 90, no. 1: 166–193.

[aur70010-bib-0007] Broadbent, J. , I. Galic , and M. A. Stokes . 2013. “Validation of Autism Spectrum Quotient Adult Version in an Australian Sample.” Autism Research and Treatment 2013: 1–7. 10.1155/2013/984205. PMC3665170 23762552

[aur70010-bib-0008] Brunet, D. , M. M. Murray , and C. M. Michel . 2011. “Spatiotemporal Analysis of Multichannel EEG: CARTOOL.” Computational Intelligence and Neuroscience 2011, no. 2: 1–15. 10.1155/2011/813870. 21253358 PMC3022183

[aur70010-bib-0009] Bylemans, T. , E. Heleven , K. Baetens , N. Deroost , C. Baeken , and F. Van Overwalle . 2023. “Mentalizing and Narrative Coherence in Autistic Adults: Cerebellar Sequencing and Prediction.” Neuroscience & Biobehavioral Reviews 146: 105045. 10.1016/j.neubiorev.2023.105045. 36646260

[aur70010-bib-0010] Cox, J. C. , D. Friedman , and S. Gjerstad . 2007. “A Tractable Model of Reciprocity and Fairness.” Games and Economic Behavior 59, no. 1: 17–45. 10.1016/j.geb.2006.05.001.

[aur70010-bib-0011] Crompton, C. J. , D. Ropar , C. V. M. Evans‐Williams , E. G. Flynn , and S. Fletcher‐Watson . 2020. “Autistic Peer‐To‐Peer Information Transfer Is Highly Effective.” Autism 24, no. 7: 1704–1712. 10.1177/1362361320919286. 32431157 PMC7545656

[aur70010-bib-0012] Davis, M. H. 1983. “Measuring Individual Differences in Empathy: Evidence for a Multidimensional Approach.” Journal of Personality and Social Psychology 44, no. 1: 113–126. 10.1037/0022-3514.44.1.113.

[aur70010-bib-0013] Davis, R. , and C. J. Crompton . 2021. “What Do New Findings About Social Interaction in Autistic Adults Mean for Neurodevelopmental Research?” Perspectives on Psychological Science 16, no. 3: 649–653. 10.1177/1745691620958010. 33560175 PMC8114326

[aur70010-bib-0014] de Lacy, N. , D. Doherty , B. H. King , S. Rachakonda , and V. D. Calhoun . 2017. “Disruption to Control Network Function Correlates With Altered Dynamic Connectivity in the Wider Autism Spectrum.” NeuroImage: Clinical 15: 513–524. 10.1016/j.nicl.2017.05.024. 28652966 PMC5473646

[aur70010-bib-0015] Eagly & Wood . 1991. “Explaining Sex Differences_Social‐Behavior_Meta‐Analytic‐Perspective.”

[aur70010-bib-0016] Ezaki, T. , Y. Himeno , T. Watanabe , and N. Masuda . 2021. “Modelling State‐Transition Dynamics in Resting‐State Brain Signals by the Hidden Markov and Gaussian Mixture Models.” European Journal of Neuroscience 54, no. 4: 5404–5416. 10.1111/ejn.15386. 34250639 PMC9291560

[aur70010-bib-0017] Faul, F. , E. Erdfelder , A.‐G. Lang , and A. Buchner . 2007. “G*Power 3: A Flexible Statistical Power Analysis Program for the Social, Behavioral, and Biomedical Sciences.” Behavior Research Methods 39: 175–191. 10.3758/BF03193146. 17695343

[aur70010-bib-0018] Fehr, E. , and K. M. Schmidt . 1999. “A Theory of Fairness, Competition, and Cooperation.” Quarterly Journal of Economics 114, no. 3: 817–868.

[aur70010-bib-0019] Feng, C. , S. B. Eickhoff , T. Li , et al. 2021. “Common Brain Networks Underlying Human Social Interactions: Evidence From Large‐Scale Neuroimaging Meta‐Analysis.” Neuroscience and Biobehavioral Reviews 126: 289–303. 10.1016/j.neubiorev.2021.03.025. 33781834

[aur70010-bib-0020] Forbes, P. A. G. , I. Chaliani , L. Schilbach , and T. Kalenscher . 2023. “Autistic Adults Show Enhanced Generosity to Socially Distant Others.” Autism 28, no. 4: 999–1009. 10.1177/13623613231190674. 37606240

[aur70010-bib-0021] Forgeot D'Arc, B. , M. Devaine , and J. Daunizeau . 2020. “Social Behavioural Adaptation in Autism.” PLoS Computational Biology 16, no. 3: 1–18. 10.1371/journal.pcbi.1007700. PMC7108744 32176684

[aur70010-bib-0022] Gernsbacher, M. A. 2006. “Toward a Behavior of Reciprocity NIH Public Access.” Journal of Developmental Processes 1, no. 1: 139–152. 25598865 PMC4296736

[aur70010-bib-0023] Gernsbacher, M. A. , and M. Yergeau . 2019. “Empirical Failures of the Claim That Autistic People Lack a Theory of Mind.” Archives of Scientific Psychology 7, no. 1: 102–118. 10.1037/arc0000067. 31938672 PMC6959478

[aur70010-bib-0024] Ghahramani, Z. , and M. J. Beai . 2000. “Variational Inference for Bayesian Mixtures of Factor Analysers.” In Advances in Neural Information Processing Systems, edited by S. A. Solla , T. K. Leen , and K. Muller , vol. 12, 449–455. MIT Press.

[aur70010-bib-0025] Gotts, S. J. , Z. S. Saad , H. J. Jo , G. L. Wallace , R. W. Cox , and A. Martin . 2013. “The Perils of Global Signal Regression for Group Comparisons: A Case Study of Autism Spectrum Disorders.” Frontiers in Human Neuroscience 7: 356. 10.3389/fnhum.2013.00356. 23874279 PMC3709423

[aur70010-bib-0026] Guo, Z. , X. Tang , S. Xiao , et al. 2024. “Systematic Review and Meta‐Analysis: Multimodal Functional and Anatomical Neural Alterations in Autism Spectrum Disorder.” Molecular Autism 15, no. 1: 16. 10.1186/s13229-024-00593-6. 38576034 PMC10996269

[aur70010-bib-0027] Hartley, C. , and S. Fisher . 2018. “Do Children With Autism Spectrum Disorder Share Fairly and Reciprocally?” Journal of Autism and Developmental Disorders 48, no. 8: 2714–2726. 10.1007/s10803-018-3528-7. 29512018 PMC6061008

[aur70010-bib-0028] Hase, A. , M. Haynes , and G. Hasler . 2023. “Using Simple Economic Games to Assess Social Orienting and Prosocial Behavior in Adolescents With Autism Spectrum Disorder.” Autism Research 16, no. 6: 1199–1209. 10.1002/aur.2931. 37057313

[aur70010-bib-0029] Hutchison, R. M. , T. Womelsdorf , E. A. Allen , et al. 2013. “Dynamic Functional Connectivity: Promise, Issues, and Interpretations.” NeuroImage 80: 360–378. 10.1016/j.neuroimage.2013.05.079. 23707587 PMC3807588

[aur70010-bib-0030] Hyatt, C. J. , B. E. Wexler , B. Pittman , et al. 2022. “Atypical Dynamic Functional Network Connectivity State Engagement During Social–Emotional Processing in Schizophrenia and Autism.” Cerebral Cortex 32, no. 16: 3406–3422. 10.1093/cercor/bhab423. 34875687 PMC9376868

[aur70010-bib-0031] Ilioska, I. , M. Oldehinkel , A. Llera , et al. 2023. “Connectome‐Wide Mega‐Analysis Reveals Robust Patterns of Atypical Functional Connectivity in Autism.” Biological Psychiatry 94, no. 1: 29–39. 10.1016/j.biopsych.2022.12.018. 36925414

[aur70010-bib-0032] Ingalhalikar, M. , A. Smith , D. Parker , et al. 2014. “Sex Differences in the Structural Connectome of the Human Brain.” Proceedings of the National Academy of Sciences 111, no. 2: 823–828. 10.1073/pnas.1316909110. PMC3896179 24297904

[aur70010-bib-0033] Jasmin, K. , S. J. Gotts , Y. Xu , et al. 2019. “Overt Social Interaction and Resting State in Young Adult Males With Autism: Core and Contextual Neural Features.” Brain 142, no. 3: 808–822. 10.1093/brain/awz003. 30698656 PMC6391610

[aur70010-bib-0034] Jasmin, K. , A. Martin , and S. J. Gotts . 2023. “Atypical Connectivity Aids Conversation in Autism.” Scientific Reports 13, no. 1: 1–8. 10.1038/s41598-023-32249-5. 37002277 PMC10066277

[aur70010-bib-0035] Jenkinson, M. , C. F. Beckmann , T. E. J. Behrens , M. W. Woolrich , and S. M. Smith . 2012. “Fsl.” NeuroImage 62, no. 2: 782–790. 10.1016/j.neuroimage.2011.09.015. 21979382

[aur70010-bib-0036] Jin, P. , Y. Wang , Y. Li , et al. 2020. “The Fair Decision‐Making of Children and Adolescents With High‐Functioning Autism Spectrum Disorder From the Perspective of Dual‐Process Theories.” BMC Psychiatry 20, no. 1: 1–11. 10.1186/s12888-020-02562-8. 32252695 PMC7137314

[aur70010-bib-0037] Klapwijk, E. T. , M. Aghajani , G.‐J. Lelieveld , et al. 2017. “Differential Fairness Decisions and Brain Responses After Expressed Emotions of Others in Boys With Autism Spectrum Disorders.” Journal of Autism and Developmental Disorders 47, no. 8: 2390–2400. 10.1007/s10803-017-3159-4. 28516421 PMC5509841

[aur70010-bib-0038] Kuhl, J. 1994. “Action Versus State Orientation: Psychometric Properties of the Action Control Scale (ACS‐90).” In Volition and Personality: Action Versus State Orientation, edited by J. Kuhl and J. Beckmann , 47–56. Hogrefe Huber.

[aur70010-bib-0039] Li, Y. , Y. Zhu , B. A. Nguchu , et al. 2020. “Dynamic Functional Connectivity Reveals Abnormal Variability and Hyper‐Connected Pattern in Autism Spectrum Disorder.” Autism Research 13, no. 2: 230–243. 10.1002/aur.2212. 31614075

[aur70010-bib-0040] Lord, C. , T. S. Brugha , T. Charman , et al. 2020. “Autism Spectrum Disorder.” Nature Reviews Disease Primers 6, no. 1: 5. 10.1038/s41572-019-0138-4. PMC8900942 31949163

[aur70010-bib-0041] Macchia, A. , L. Albantakis , P. T. Zebhauser , M. L. Brandi , L. Schilbach , and A. K. Brem . 2024. “Autistic Adults Avoid Unpredictability in Decision‐Making.” Journal of Autism and Developmental Disorders: 1–13. 10.1007/s10803-024-06503-2. PMC12589206 39158770

[aur70010-bib-0042] Maliske, L. , and P. Kanske . 2022. “The Social Connectome – Moving Toward Complexity in the Study of Brain Networks and Their Interactions in Social Cognitive and Affective Neuroscience.” Frontiers in Psychiatry 13, no. April: 1–7. 10.3389/fpsyt.2022.845492. PMC9016142 35449570

[aur70010-bib-0043] Mash, L. E. , A. C. Linke , L. A. Olson , I. Fishman , T. T. Liu , and R. Müller . 2019. “Transient States of Network Connectivity Are Atypical in Autism: A Dynamic Functional Connectivity Study.” Human Brain Mapping 40, no. 8: 2377–2389. 10.1002/hbm.24529. 30681228 PMC6549695

[aur70010-bib-0044] Maurer, C. , V. Chambon , S. Bourgeois‐Gironde , M. Leboyer , and T. Zalla . 2018. “The Influence of Prior Reputation and Reciprocity on Dynamic Trust‐Building in Adults With and Without Autism Spectrum Disorder.” Cognition 172: 1–10. 10.1016/j.cognition.2017.11.007. 29197230

[aur70010-bib-0045] Milton, D. E. M. 2012. “On the Ontological Status of Autism: The Double Empathy Problem.” Disability & Society 27, no. 6: 883–887. 10.1080/09687599.2012.710008.

[aur70010-bib-0046] Misaki, M. , K. L. Kerr , E. L. Ratliff , et al. 2021. “Beyond Synchrony: The Capacity of fMRI Hyperscanning for the Study of Human Social Interaction.” Social Cognitive and Affective Neuroscience 16, no. 1–2: 84–92. 10.1093/scan/nsaa143. 33104783 PMC7812622

[aur70010-bib-0047] Molins, F. , N. Ben‐Hassen Jemni , D. Garrote‐Petisco , and M. Á. Serrano . 2024. “Highly Logical and Non‐emotional Decisions in Both Risky and Social Contexts: Understanding Decision Making in Autism Spectrum Disorder Through Computational Modeling.” Cognitive Processing 25, no. 3: 0123456789. 10.1007/s10339-024-01182-4. PMC11269346 38526667

[aur70010-bib-0048] Nunes, A. S. , N. Peatfield , V. Vakorin , and S. M. Doesburg . 2019. “Idiosyncratic Organization of Cortical Networks in Autism Spectrum Disorder.” NeuroImage 190: 182–190. 10.1016/j.neuroimage.2018.01.022. 29355768

[aur70010-bib-0049] Backer van Ommeren, T. , M. Vreugdenhil , H. M. Koot , et al. 2022. “A New Real‐Life Test for Reciprocity in Autistic Adults: The Interactive Drawing Test.” Frontiers in Psychiatry 13: 1–10. 10.3389/fpsyt.2022.842902. PMC8977513 35386524

[aur70010-bib-0050] Pan, H. , Y. Mao , P. Liu , et al. 2023. “Extracting Transition Features Among Brain States Based on Coarse‐Grained Similarity Measurement for Autism Spectrum Disorder Analysis.” Medical Physics 50, no. 10: 6269–6282. 10.1002/mp.16406. 36995984

[aur70010-bib-0051] Peng, X. , T. Li , G. Liu , W. Ni , and L. Yi . 2024. “Enhanced Neural Synchronization During Social Communications Between Dyads With High Autistic Traits.” Cerebral Cortex 34, no. 13: 104–111. 10.1093/cercor/bhae027. 38696603

[aur70010-bib-0052] Proverbio, A. M. 2023. “Sex Differences in the Social Brain and in Social Cognition.” Journal of Neuroscience Research 101, no. 5: 730–738. 10.1002/jnr.24787. 33608982

[aur70010-bib-0053] Quiñones‐Camacho, L. E. , F. A. Fishburn , K. Belardi , D. L. Williams , T. J. Huppert , and S. B. Perlman . 2021. “Dysfunction in Interpersonal Neural Synchronization as a Mechanism for Social Impairment in Autism Spectrum Disorder.” Autism Research 14, no. 8: 1585–1596. 10.1002/aur.2513. 33847461 PMC11413982

[aur70010-bib-0054] Rabany, L. , S. Brocke , V. D. Calhoun , et al. 2019. “Dynamic Functional Connectivity in Schizophrenia and Autism Spectrum Disorder: Convergence, Divergence and Classification.” NeuroImage: Clinical 24: 101966. 10.1016/j.nicl.2019.101966. 31401405 PMC6700449

[aur70010-bib-0055] Redcay, E. , and L. Schilbach . 2019. “Using Second‐Person Neuroscience to Elucidate the Mechanisms of Social Interaction.” Nature Reviews Neuroscience 20, no. 8: 495–505. 10.1038/s41583-019-0179-4. 31138910 PMC6997943

[aur70010-bib-0056] Rifai, O. M. , S. Fletcher‐Watson , L. Jiménez‐Sánchez , and C. J. Crompton . 2022. “Investigating Markers of Rapport in Autistic and Nonautistic Interactions.” Autism in Adulthood 4, no. 1: 3–11. 10.1089/aut.2021.0017. 36600904 PMC8992924

[aur70010-bib-0057] Roy, D. , and L. Q. Uddin . 2021. “Atypical Core‐Periphery Brain Dynamics in Autism.” Network Neuroscience 5, no. 2: 295–321. 10.1162/netn_a_00181. 34189366 PMC8233106

[aur70010-bib-0058] Sally, D. , and E. Hill . 2006. “The Development of Interpersonal Strategy: Autism, Theory‐of‐Mind, Cooperation and Fairness.” Journal of Economic Psychology 27, no. 1: 73–97. 10.1016/j.joep.2005.06.015.

[aur70010-bib-0059] Santamaría‐García, H. , S. Baez , C. Gómez , et al. 2020. “The Role of Social Cognition Skills and Social Determinants of Health in Predicting Symptoms of Mental Illness.” Translational Psychiatry 10, no. 1: 165. 10.1038/s41398-020-0852-4. 32513944 PMC7280528

[aur70010-bib-0060] Schaefer, A. , R. Kong , E. M. Gordon , et al. 2018. “Local‐Global Parcellation of the Human Cerebral Cortex From Intrinsic Functional Connectivity MRI.” Cerebral Cortex 28, no. 9: 3095–3114. 10.1093/cercor/bhx179. 28981612 PMC6095216

[aur70010-bib-0061] Schilbach, L. 2016. “Towards a Second‐Person Neuropsychiatry.” Philosophical Transactions of the Royal Society, B: Biological Sciences 371, no. 1686: 20150081. 10.1098/rstb.2015.0081 PMC4685526 26644599

[aur70010-bib-0062] Schopler, E. , M. Van Bourgondien , J. Wellman , and S. Love . 2010. Childhood Autism Rating Scale – Second Edition (CARS2): Manual. Western Psychological Services.

[aur70010-bib-0063] Schurz, M. , L. Maliske , and P. Kanske . 2020. “Cross‐Network Interactions in Social Cognition: A Review of Findings on Task Related Brain Activation and Connectivity.” Cortex 130: 142–157. 10.1016/j.cortex.2020.05.006. 32653744

[aur70010-bib-0064] Shamay‐Tsoory, S. G. , and A. Mendelsohn . 2019. “Real‐Life Neuroscience: An Ecological Approach to Brain and Behavior Research.” Perspectives on Psychological Science 14, no. 5: 841–859. 10.1177/1745691619856350. 31408614

[aur70010-bib-0065] Shaw, D. , K. Czekóová , M. Gajdoš , R. Staněk , J. Špalek , and M. Brázdil . 2019. “Social Decision‐Making in the Brain: Input‐State‐Output Modelling Reveals Patterns of Effective Connectivity Underlying Reciprocal Choices.” Human Brain Mapping 40, no. 2: 699–712. 10.1002/hbm.24446. 30431199 PMC6587762

[aur70010-bib-0066] Shaw, D. J. , K. Czekóová , R. Mareček , H. B. Špiláková , and M. Brázdil . 2023. “The Interacting Brain: Dynamic Functional Connectivity Among Canonical Brain Networks Dissociates Cooperative From Competitive Social Interactions.” NeuroImage 269: 119933. 10.1016/j.neuroimage.2023.119933. 36754124

[aur70010-bib-0067] Shaw, D. J. , K. Czekóová , R. Staněk , et al. 2018. “A Dual‐fMRI Investigation of the Iterated Ultimatum Game Reveals That Reciprocal Behaviour Is Associated With Neural Alignment.” Scientific Reports 8, no. 1: 1–13. 10.1038/s41598-018-29233-9. 30022087 PMC6051991

[aur70010-bib-0068] Taghia, J. , W. Cai , S. Ryali , et al. 2018. “Uncovering Hidden Brain State Dynamics That Regulate Performance and Decision‐Making During Cognition.” Nature Communications 9, no. 1: 2505. 10.1038/s41467-018-04723-6. PMC6021386 29950686

[aur70010-bib-0069] Tei, S. , J. Fujino , R. I. Hashimoto , et al. 2018. “Inflexible Daily Behaviour Is Associated With the Ability to Control an Automatic Reaction in Autism Spectrum Disorder.” Scientific Reports 8, no. 1: 4–9. 10.1038/s41598-018-26465-7. 29795394 PMC5967343

[aur70010-bib-0070] Thaler, H. , L. Albantakis , and L. Schilbach . 2024. “Social Cognitive and Interactive Abilities in Autism.” In Oxford Handbook of Developmental Cognitive Neuroscience, edited by K. C. Kadosh . 10.1093/oxfordhb/9780198827474.013.29.

[aur70010-bib-0071] Thomas Yeo, B. T. , F. M. Krienen , J. Sepulcre , et al. 2011. “The Organization of the Human Cerebral Cortex Estimated by Intrinsic Functional Connectivity.” Journal of Neurophysiology 106, no. 3: 1125–1165. 10.1152/jn.00338.2011. 21653723 PMC3174820

[aur70010-bib-0072] Trovato, A. N. 2019. Social Decision‐Making in Individuals With Autism Spectrum Disorder: Examining the Effect of Facial Expression on Ultimatum Game Decisions. Indiana University of Pennsylvania.

[aur70010-bib-0073] Uddin, L. Q. , B. T. T. Yeo , and R. N. Spreng . 2019. “Towards a Universal Taxonomy of Macro‐Scale Functional Human Brain Networks.” Brain Topography 32, no. 6: 926–942. 10.1007/s10548-019-00744-6. 31707621 PMC7325607

[aur70010-bib-0074] Velikonja, T. , A.‐K. Fett , and E. Velthorst . 2019. “Patterns of Nonsocial and Social Cognitive Functioning in Adults With Autism Spectrum Disorder: A Systematic Review and Meta‐Analysis.” JAMA Psychiatry 76, no. 2: 135–151. 10.1001/jamapsychiatry.2018.3645. 30601878 PMC6439743

[aur70010-bib-0075] Wadge, H. , R. Brewer , G. Bird , I. Toni , and A. Stolk . 2019. “Communicative Misalignment in Autism Spectrum Disorder.” Cortex 115: 15–26. 10.1016/j.cortex.2019.01.003. 30738998

[aur70010-bib-0076] Wager, T. D. , and T. E. Nichols . 2003. “Optimization of Experimental Design in fMRI: A General Framework Using a Genetic Algorithm.” NeuroImage 18, no. 2: 293–309. 10.1016/S1053-8119(02)00046-0. 12595184

[aur70010-bib-0077] Wang, M. , L. Wang , B. Yang , et al. 2022. “Disrupted Dynamic Network Reconfiguration of the Brain Functional Networks of Individuals With Autism Spectrum Disorder.” Brain Communications 4, no. 4: fcac177. 10.1093/braincomms/fcac177. 35950094 PMC9356733

[aur70010-bib-0078] Wang, Y. , Y. Xiao , Y. Li , et al. 2019. “Exploring the Relationship Between Fairness and ‘Brain Types’ in Children With High‐Functioning Autism Spectrum Disorder.” Progress in Neuro‐Psychopharmacology & Biological Psychiatry 88: 151–158. 10.1016/j.pnpbp.2018.07.008. 30009870

[aur70010-bib-0079] Watanabe, T. , and G. Rees . 2017. “Brain Network Dynamics in High‐Functioning Individuals With Autism.” Nature Communications 8: 1–14. 10.1038/ncomms16048. PMC5504272 28677689

[aur70010-bib-0080] Wheatley, T. , A. Boncz , I. Toni , and A. Stolk . 2019. “Beyond the Isolated Brain: The Promise and Challenge of Interacting Minds.” Neuron 103, no. 2: 186–188. 10.1016/j.neuron.2019.05.009. 31319048 PMC7789915

[aur70010-bib-0081] Woodcock, K. A. , C. Cheung , D. González Marx , and W. Mandy . 2020. “Social Decision Making in Autistic Adolescents: The Role of Theory of Mind, Executive Functioning and Emotion Regulation.” Journal of Autism and Developmental Disorders 50, no. 7: 2501–2512. 10.1007/s10803-019-03975-5. 30879258

[aur70010-bib-0082] Yeshurun, Y. , M. Nguyen , and U. Hasson . 2021. “The Default Mode Network: Where the Idiosyncratic Self Meets the Shared Social World.” Nature Reviews Neuroscience 22, no. 3: 181–192. 10.1038/s41583-020-00420-w. 33483717 PMC7959111

[aur70010-bib-0083] Yue, X. , G. Zhang , X. Li , et al. 2022. “Abnormal Dynamic Functional Network Connectivity in Adults With Autism Spectrum Disorder.” Clinical Neuroradiology 32, no. 4: 1087–1096. 10.1007/s00062-022-01173-y. 35543744

[aur70010-bib-0084] Zhang, J. , Z. Huang , S. Tumati , and G. Northoff . 2020. “Rest‐Task Modulation of fMRI‐Derived Global Signal Topography Is Mediated by Transient Coactivation Patterns.” PLoS Biology 18, no. 7: e3000733. 10.1371/journal.pbio.3000733. 32649707 PMC7375654

[aur70010-bib-0085] Zhang, J. , and G. Northoff . 2022. “Beyond Noise to Function: Reframing the Global Brain Activity and Its Dynamic Topography.” Communications Biology 5, no. 1: 1350. 10.1038/s42003-022-04297-6. 36481785 PMC9732046

[aur70010-bib-0086] Zhuang, W. , H. Jia , Y. Liu , et al. 2023. “Identification and Analysis of Autism Spectrum Disorder via Large‐Scale Dynamic Functional Network Connectivity.” Autism Research 16, no. 8: 1512–1526. 10.1002/aur.2974. 37365978

