# Supplementary material for: Altered Patterns of Dynamic Functional Connectivity Underpin Reduced Expressions of Social–Emotional Reciprocity in Autistic Adults
Source: Autism Res. 2025 Feb 24;18(4):725–40. doi: 10.1002/aur.70010 (PMC12015814; doi:10.1002/aur.70010)
Supplement: Supplementary file 1 — Data S1. Supporting Information. [file AUR-18-725-s001.docx]

**Title**

**Altered patterns of dynamic functional connectivity underpin reduced expressions of social-emotional reciprocity in autistic adults.**

**Authors**

Czekóová, K., Mareček, R., Staněk, R., Hartley, C., Kessler, K., Hlavatá, P., Ošlejšková, H., Brázdil, M., & Shaw, D. J.

**Participant Instructions**

*(Note: Czech-to-English translation)*

You are taking part in an economic experiment where you can earn money depending on your decisions. Therefore, please read the following instructions carefully.

Do not hesitate to ask us any questions you may have after reading these instructions. It is very important that you understand all the rules before you begin the experiment.

At the beginning of the experiment, you and your partner will be assigned randomly to one of two roles: One of you will be assigned to the role of **"Proposer"** (whose colour is blue) and the other will become the **"Responder"** (whose colour is red). This role assignment will not change during the experiment.

The course of the round

At the start of every round the **Proposer** is given **100Kč**, and their task is to divide this between themselves and the Responder. The Proposer will have a selection of two options for dividing the sum of money and will have 4 seconds to choice one division to offer. They will press **the left button** **to select the division on the left side and the right button to select the division on the right side.**


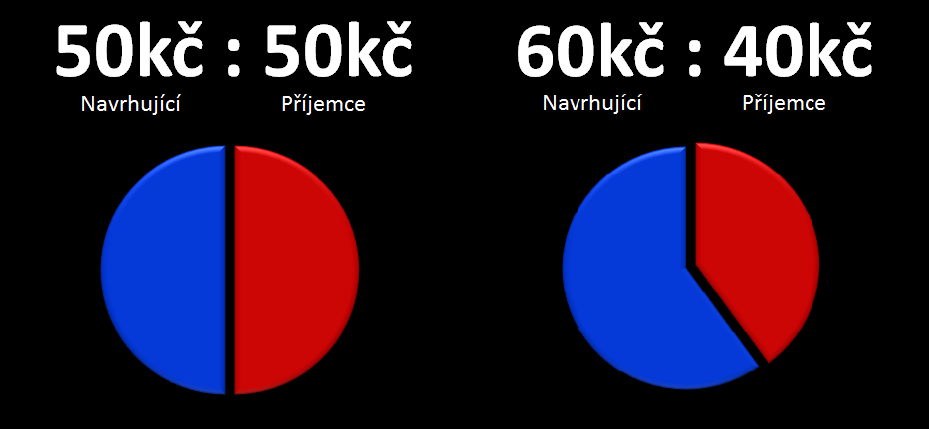


*Example. In the figure depicted above, the Proposer is asked to divide the money using a ratio of 50Kč for themselves and 50Kč for the Responder, or 60Kč for themselves and 40Kč for the Responder. The Proposer has to make their choice within 4 seconds, and the options are displayed throughout this period. If the Proposer presses the left button response to the above stimulus, the 50:50 division will be offered; a press of the right button will mean the division of 60:40 will be offered.*

The **Responder** will always see the two divisions from which the Proposer makes their selection but cannot influence this selection in any way. After 4 seconds, the division selected by the Proposer will be framed in yellow. The Responder will then decide whether to accept or reject the proposed division. If they decide to **accept** it, it will be considered to be an agreement between the players and the money will be distributed according to the proposed division. If the Responder **rejects** the amount, both the Responder and the Proposer receive **0Kč**. The Responder has to make a choice whether to accept or reject the division within 4 seconds of the proposed division being highlighted. If they press **the left button, the division will be accepted;** by pressing the **right button they will reject the offer**.

The divisions offered in each round have been predetermined and are therefore not dependent on the choices you make during the experiment.

**IMPORTANT! If the Proposer or Responder does not make a choice within the specified time limit, the round ends and both players get 0Kč.** The outcomes of other rounds will not be influenced by this in any way, however.

These monetary exchanges will be randomly mixed among rounds in which the Proposer selects a division of colour from two semicircles. The Responder can again decide whether or not to accept the proposed offer of colour. **These decisions do not influence the financial compensation; they have no effect on overall winnings or performance in the game.**


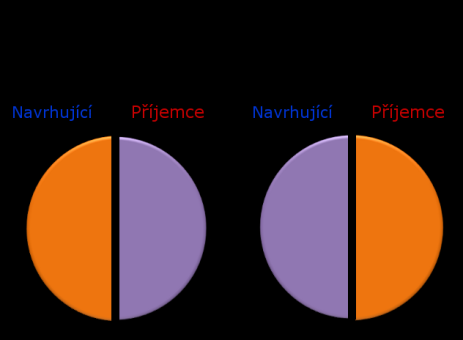


Number of rounds

The rounds described above will be repeated over the course of the experiment. The number of repetitions will not be announced in advance, but the duration of the experiment can be expected to be around 60-90 minutes.

Reward
At the end of the experiment, **3 rounds will be selected randomly.** You will earn your combined reward across these rounds.

**Choice sets**

| **Condition** | **Choice Sets** | **Payoff Difference** |
| --- | --- | --- |
| **PP** | 70:30\|55:45 | 15 |
|  | 70:30\|60:40 | 10 |
|  | 65:35\|55:45 | 10 |
|  | 65:35\|60:40 | 5 |
|  | 60:40\|55:45 | 5 |
| **PR** | 70:30\|30:70 | 40 |
|  | 65:35\|35:65 | 30 |
|  | 65:35\|45:55 | 20 |
|  | 60:40\|40:60 | 20 |
|  | 55:45\|45:55 | 10 |

**Supplementary Table S1.** Choice sets. Underlined values represent the payoff to the Proposer in each of the two constituent options. The right-most option presents the “fair” division with minimal advantageous (PP) or disadvantageous inequity (PR), and the alternative option presents the “unfair” division. The difference in each player’s payoff between the fair and unfair division is shown beside each choice set. Each of the 10 choice sets was presented 6 times throughout the experiment, with the unfair division on the left or right for an equal number of rounds. PP = Proposer-Proposer condition, PR = Proposer-Responder condition.

**MRI Data Acquisition**

Structural and functional brain images were acquired from both the Proposer and Responder of a given pair simultaneously with one of two identical 3T Siemens Prisma scanners equipped with a 64-channel bird-cage head coil. For both sets of dyads, a high-resolution T1-weighted anatomical image was acquired first (MPRAGE, TR/TE = 2300/2.3 ms; flip angle = 8°; matrix = 240×224×224, 1 mm3 voxels) followed by blood-oxygen-level-dependent (BOLD) images acquired with a T2*-weighted echo-planar imaging sequence with parallel acquisition (i-PAT; GRAPPA acceleration factor = 2; 34 axial slices; TR/TE = 2000/35ms; flip angle = 60°; matrix = 68×68×34, 3×3×4 mm3 voxels). Axial slices were acquired in interleaved order, oriented to a line connecting the base of the cerebellum to the base of orbitofrontal cortex for whole-brain coverage. Functional imaging was performed in a single run comprising 690 volumes (23 min), with three dummy volumes acquired at the beginning to allow the gradients to reach steady state. An external programmable signal generator (Siglent SDG1025, [www.siglent.com](http://www.siglent.com)) initiated synchronous acquisition sequences in both scanners.

**MRI Data Pre-processing**

MRI data from both sets of dyads were pre-processed in the exact same way using various tools packaged within FMRIB’s software library (FSL; Jenkinson et al., 2012). Slice-time correction was achieved with Fourier-space time-series phase-shifting. Using FEAT v6.00, each time-series was high-pass filtered to remove low-frequency drifts (Gaussian-weighted least-squares straight line fitting; sigma = 60s), and spatially smoothed using a 5 mm full-width half-maximum Gaussian kernel. The time-series were intensity normalised using grand-mean scaling of the entire 4D dataset by a single multiplicative factor, minimising any unspecific time effects. Initial motion correction of the BOLD time-series was performed with MCFLIRT. To identify any signal related to residual motion or physiological noise, we performed a subsequent probabilistic independent component analysis using MELODIC (Beckmann, 2012). This decomposed the time-series into 50 independent spatial and temporal components, and those capturing artifactual signals were identified automatically with the Spatially Organized Component Klassifikator (Bhaganagarapu et al., 2013). Signals relating to these noise components were then regressed out of the time-series. For each participant, the anatomical image was registered linearly (12 DOF) to the MNI template with FLIRT and this affine registration was used subsequently to register the image non-linearly to the standard template using FNIRT. Again using FNIRT, the functional time-series was normalised non-linearly to the MNI template using the *applywarp* command. The functional time-series extracted from each of the 400 parcels was despiked using a Wavelet-based algorithm (Patel et al., 2014) and bandpass filtered at 0.01 to 0.15 Hz.

**Reciprocity Parameter Estimation**

***Reciprocity Model***

On each round, the expected utility of each monetary division was specified as follows:

1. $U(x,100-x)=x+(\theta+ \epsilon)(100-x)$

In Equation (1), $x$ is the player’s portion of the division, $\theta$ is a scalar that represents their emotional state, and $\epsilon$ is a random variable with standard logistic distribution that adds stochasticity to behaviour (e.g., unintended responses) and thus represents an unobserved component of the utility function.

The emotional state was formulated as:

1. $\theta=\alpha_{i}(x-x_{0})$

Equation (2) incorporates a time-invariant, player-specific reciprocity parameter ($\alpha_{i}$) that weights a comparison of the player’s share ($x$) against a fairness reference point ($x_{0}$) by the extent to which a player’s choices are influenced by their partner’s prior behaviour. The reference point, ${(x}_{0}$) is different for each choice set and therefore changes on each round.

This utility function was used to model round-by-round expected utility for both players. The Responder accepts a proposal when:

1. $x+\left( \theta+ \epsilon\right)\left( 100-x \right)>0$

The Proposer offers more generous division when:

1. $P_{1}\left( x_{1}+\left( \theta+\epsilon\right)(100-x_{1}) \right)> P_{2}(x_{2}+\left( \theta+\epsilon\right)(100-x_{2}))$

From the Proposer’s perspective, $x_{1}$ and $x_{2}$ represent the division with minimal (or disadvantageous) and maximal advantageous inequity, respectively. *P_i_* represents the probability that the Responder will accept a division given their prior behaviour. The Proposer thus makes an offer that benefits themselves maximally only if they believe the offer is likely to be accepted. As we assume that *ϵ* has a logistic distribution, the probability of the Responder accepting an offer can be expressed as the value of the cumulative distribution function of the logistic distribution:

1. $F\left( z \right)=e^{z}\left( e^{z}+1 \right)^{-1}$ $F\left( z \right)=e^{z}\left( e^{z}+1 \right)^{-1}$

This is evaluated at:

1. $z=x/{(100-x)}+\alpha_{i} x-\alpha_{i}x_{0}$ $z=x/{(100-x)}+\alpha_{i} x-\alpha_{i}x_{0}$

Responders’ α and $x_{0}$ parameters are estimated by maximizing the log-likelihood function:

1. $\ln L=\sum_{i} \sum_{j} y_{ij}\ln F\left( z \right)+\left( 1-y_{ij} \right)\ln\left( 1-F\left( z \right) \right)$

Variable $y_{ij}$ is binary variable, taking a value of one if Responder *i* in round *j* accepted the offer and zero otherwise. For Proposers, after some algebraic manipulations it can be shown that the probability of offering the division with minimal advantageous inequity (MIN offers) is given by the value of the cumulative distribution function of the logistic distribution $F\left( v \right)$:

1. $v=\frac{P_{1}x_{1}-P_{2}x_{2}}{P_{1}\left( 100-x_{1} \right)-P_{2}\left( 100-x_{2} \right)}+\frac{\alpha_{i}(P_{1}x_{1}\left( 100-x_{1} \right)-P_{2}x_{2}\left( 100-x_{2} \right))}{P_{1}\left( 100-x_{1} \right)-P_{2}\left( 100-x_{2} \right)}-\alpha_{i}x_{0}$

Estimation of the Proposer’s α and $x_{0}$ parameters is complicated by the fact that the probability of making the MIN offer depends upon the expected decision of the Responder. To estimate the expected probability of acceptance on a given round, we considered the Responder’s previous behaviour; specifically, using the procedure described above we estimate the Responder’s parameters α and $x_{0}$ using only the last *M* rounds (the *memory* parameter). The memory parameter therefore represents the range of preceding Responder choices over which the Proposer’s prediction of their opponent’s upcoming response is maximised. The estimated value of parameters α and $x_{0}$ determine the probability that the Responder will accept a particular offer via the relationship $P_{i}=F(z)$. Given these probabilities of acceptance, the Proposer’s parameters α and $x_{0}$ are estimated by maximizing the log-likelihood function:

1. $\ln L=\sum_{i} \sum_{j} y_{ij}\ln F\left( v \right)+\left( 1-y_{ij} \right)\ln\left( 1-F\left( v \right) \right)$

Again, $y_{ij}$ is a binary variable taking a value of one if the Proposer *i* in period *j* makes the more generous offer and zero otherwise. The value of *M* was based upon the fit to the actual Proposer’s behaviour; namely, acceptance probabilities were first estimated on the basis of the Responder’s decisions over all possible ranges of preceding UG rounds (2-119), and the Proposer’s α and $x_{0}$ parameters were then re-estimated with every possible range *M*. The optimal range was defined as the best-fitting model by log-likelihood. The *memory* parameter was estimated only for Proposers because their payoff on a given round depends upon the expected (unknown) decision of the Responder. In contrast, the round-by-round payoff for the Responder depends upon the Proposer’s offer, which is known.

**
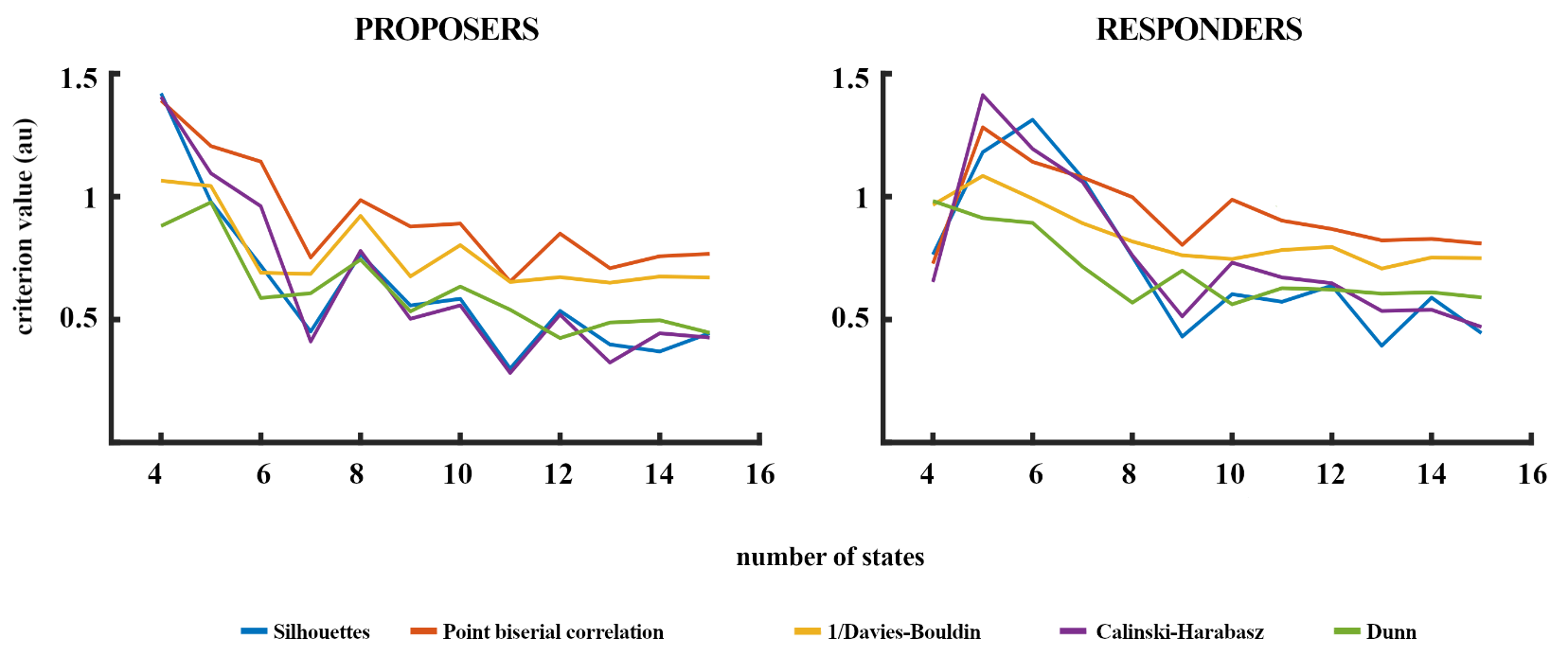
**

**Supplementary Figure S1.** Identifying the optimal number of latent brain states**.** A four-state solution was selected for both Proposers (*left*) and a five-state solution for Responders (*right*) based on the median optimal value emerging from several individual criteria.

**Supplementary Table S2.** Brain-behaviour associations for autistic Responders from AA/NA dyads.

|  |  | **Reciprocity** | | | **AQ** | | | **CARS 2** | | |
| --- | --- | --- | --- | --- | --- | --- | --- | --- | --- | --- |
|  |  | *ρ* | LCI | UCI | *ρ* | LCI | UCI | *ρ* | LCI | UCI |
| **Reciprocity** |  | 1.000 | -- | -- | -0.102 | -0.58 | 0.43 | 0.025 | -0.49 | 0.53 |
| **AQ** |  | -0.102 | -0.58 | 0.43 | 1.000 | -- | -- | 0.381 | -0.16 | 0.75 |
| **CARS 2** |  | 0.025 | -0.49 | 0.53 | 0.381 | -0.16 | 0.75 | 1.000 | -- | -- |
| **Coverage** | S1 | 0.062 | -0.46 | 0.55 | -0.261 | -0.68 | 0.28 | 0.179 | -0.36 | 0.63 |
|  | S2 | -0.118 | -0.59 | 0.42 | -0.187 | -0.63 | 0.35 | 0.269 | -0.28 | 0.68 |
|  | S3 | 0.103 | -0.43 | 0.58 | 0.325 | -0.22 | 0.72 | 0.018 | -0.49 | 0.52 |
|  | S4 | -0.126 | -0.60 | 0.41 | 0.099 | -0.43 | 0.58 | -0.157 | -0.62 | 0.38 |
|  | S5 | 0.324 | -0.22 | 0.71 | 0.325 | -0.22 | 0.72 | -0.129 | -0.60 | 0.41 |
| **Occurrence** | S1 | 0.062 | -0.46 | 0.55 | -0.178 | -0.63 | 0.36 | 0.256 | -0.29 | 0.68 |
|  | S2 | -0.218 | -0.65 | 0.33 | -0.068 | -0.56 | 0.46 | 0.201 | -0.34 | 0.64 |
|  | S3 | -0.006 | -0.51 | 0.50 | 0.385 | -0.15 | 0.75 | 0.163 | -0.38 | 0.62 |
|  | S4 | -0.205 | -0.65 | 0.34 | 0.100 | -0.43 | 0.58 | -0.091 | -0.57 | 0.44 |
|  | S5 | 0.165 | -0.37 | 0.62 | 0.400 | -0.14 | 0.76 | 0.193 | -0.35 | 0.64 |
| **Lifetime** | S1 | 0.224 | -0.32 | 0.66 | **-0.510*** | **-0.81** | **-0.004** | -0.032 | -0.53 | 0.48 |
|  | S2 | 0.224 | -0.32 | 0.66 | -0.387 | -0.75 | 0.15 | 0.109 | -0.42 | 0.59 |
|  | S3 | 0.215 | -0.33 | 0.65 | -0.242 | -0.67 | 0.30 | -0.261 | -0.68 | 0.28 |
|  | S4 | -0.100 | -0.58 | 0.43 | 0.046 | -0.47 | 0.54 | -0.230 | -0.66 | 0.31 |
|  | S5 | 0.153 | -0.39 | 0.61 | -0.102 | -0.58 | 0.43 | -0.406 | -0.76 | 0.13 |
| **Transitions** | S1→S1 | 0.210 | -0.33 | 0.65 | -0.349 | -0.73 | 0.19 | 0.037 | -0.48 | 0.54 |
|  | S2→S1 | -0.174 | -0.63 | 0.37 | -0.231 | -0.66 | 0.31 | 0.166 | -0.37 | 0.62 |
|  | S3→S1 | 0.231 | -0.31 | 0.66 | 0.066 | -0.46 | 0.56 | 0.141 | -0.40 | 0.61 |
|  | S4→S1 | 0.074 | -0.45 | 0.56 | -0.243 | -0.67 | 0.30 | -0.105 | -0.58 | 0.43 |
|  | S5→S1 | 0.257 | -0.29 | 0.68 | 0.092 | -0.44 | 0.57 | 0.366 | -0.17 | 0.74 |
|  | S1→S2 | -0.046 | -0.54 | 0.47 | -0.136 | -0.60 | 0.40 | 0.082 | -0.44 | 0.57 |
|  | S2→S2 | -0.006 | -0.51 | 0.50 | -0.301 | -0.70 | 0.24 | 0.183 | -0.36 | 0.63 |
|  | S3→S2 | -0.059 | -0.55 | 0.46 | 0.083 | -0.44 | 0.57 | 0.274 | -0.27 | 0.69 |
|  | S4→S2 | **-0.534*** | **-0.82** | **-0.04** | -0.375 | -0.74 | 0.16 | -0.181 | -0.63 | 0.36 |
|  | S5→S2 | -0.108 | -0.58 | 0.42 | 0.075 | -0.45 | 0.56 | 0.459 | -0.06 | 0.78 |
|  | S1→S3 | 0.334 | -0.21 | 0.72 | 0.101 | -0.43 | 0.58 | 0.281 | -0.26 | 0.69 |
|  | S2→S3 | -0.180 | -0.63 | 0.36 | 0.059 | -0.46 | 0.55 | 0.231 | -0.31 | 0.66 |
|  | S3→S3 | 0.233 | -0.31 | 0.66 | 0.106 | -0.43 | 0.58 | -0.113 | -0.59 | 0.42 |
|  | S4→S3 | -0.228 | -0.66 | 0.32 | 0.111 | -0.42 | 0.59 | -0.238 | -0.67 | 0.31 |
|  | S5→S3 | 0.072 | -0.45 | 0.56 | 0.062 | -0.46 | 0.55 | 0.174 | -0.37 | 0.63 |
|  | S1→S4 | -0.058 | -0.55 | 0.46 | **-0.513*** | **-0.81** | **-0.01** | 0.079 | -0.45 | 0.56 |
|  | S2→S4 | -0.320 | -0.71 | 0.22 | -0.260 | -0.68 | 0.29 | -0.063 | -0.55 | 0.46 |
|  | S3→S4 | -0.212 | -0.65 | 0.33 | 0.057 | -0.46 | 0.55 | 0.121 | -0.41 | 0.59 |
|  | S4→S4 | -0.181 | -0.63 | 0.36 | 0.105 | -0.43 | 0.58 | -0.199 | -0.64 | 0.34 |
|  | S5→S4 | -0.071 | -0.56 | 0.45 | 0.173 | -0.37 | 0.63 | -0.275 | -0.69 | 0.27 |
|  | S1→S5 | 0.089 | -0.44 | 0.57 | -0.038 | -0.54 | 0.48 | 0.261 | -0.29 | 0.68 |
|  | S2→S5 | 0.127 | -0.41 | 0.60 | 0.012 | -0.50 | 0.52 | 0.212 | -0.33 | 0.65 |
|  | S3→S5 | -0.152 | -0.61 | 0.39 | 0.336 | -0.21 | 0.72 | -0.151 | -0.61 | 0.39 |
|  | S4→S5 | -0.136 | -0.60 | 0.40 | 0.146 | -0.39 | 0.61 | 0.036 | -0.48 | 0.53 |
|  | S5→S5 | 0.232 | -0.31 | 0.66 | 0.168 | -0.37 | 0.62 | -0.266 | -0.68 | 0.28 |

***Note.*** AQ = Autism Spectrum Quotient. CARS 2 = Childhood Autism Rating Scale. S1-S5 = State 1 – State 5. *ρ* = Spearman correlation coefficient. L/UCI = lower/upper 95% confidence intervals. * *p* < .05. ** *p* < .01.

**Supplementary Table S3.** Brain-behaviour associations for non-autistic Responders from NA/NA dyads.

|  |  | **Reciprocity** | | |
| --- | --- | --- | --- | --- |
|  |  | *ρ* | LCI | UCI |
| **Reciprocity** |  | 1.000 | -- | -- |
| **Coverage** | S1 | 0.032 | -0.44 | 0.49 |
|  | S2 | 0.428 | -0.05 | 0.75 |
|  | S3 | 0.230 | -0.26 | 0.63 |
|  | S4 | -0.283 | -0.66 | 0.21 |
|  | S5 | -0.176 | -0.59 | 0.32 |
| **Occurrence** | S1 | -0.020 | -0.48 | 0.45 |
|  | S2 | 0.190 | -0.30 | 0.60 |
|  | S3 | 0.326 | -0.17 | 0.69 |
|  | S4 | -0.168 | -0.59 | 0.32 |
|  | S5 | -0.071 | -0.52 | 0.41 |
| **Lifetime** | S1 | 0.111 | -0.37 | 0.55 |
|  | S2 | **0.539*** | **0.10** | **0.80** |
|  | S3 | 0.051 | -0.43 | 0.51 |
|  | S4 | -0.156 | -0.58 | 0.33 |
|  | S5 | -0.132 | -0.56 | 0.36 |
| **Transitions** | S1→S1 | 0.071 | -0.41 | 0.52 |
|  | S2→S1 | -0.087 | -0.53 | 0.40 |
|  | S3→S1 | 0.269 | -0.23 | 0.65 |
|  | S4→S1 | -0.344 | -0.70 | 0.14 |
|  | S5→S1 | 0.083 | -0.40 | 0.53 |
|  | S1→S2 | 0.158 | -0.33 | 0.58 |
|  | S2→S2 | 0.454 | -0.02 | 0.76 |
|  | S3→S2 | 0.193 | -0.30 | 0.60 |
|  | S4→S2 | -0.139 | -0.57 | 0.35 |
|  | S5→S2 | -0.192 | -0.60 | 0.30 |
|  | S1→S3 | 0.341 | -0.15 | 0.70 |
|  | S2→S3 | -0.015 | -0.48 | 0.45 |
|  | S3→S3 | 0.038 | -0.44 | 0.50 |
|  | S4→S3 | 0.064 | -0.41 | 0.52 |
|  | S5→S3 | 0.212 | -0.28 | 0.62 |
|  | S1→S4 | **-0.660**** | **-0.86** | **-0.28** |
|  | S2→S4 | 0.308 | -0.18 | 0.68 |
|  | S3→S4 | 0.032 | -0.44 | 0.49 |
|  | S4→S4 | -0.181 | -0.60 | 0.31 |
|  | S5→S4 | -0.178 | -0.59 | 0.31 |
|  | S1→S5 | -0.227 | -0.63 | 0.27 |
|  | S2→S5 | -0.061 | -0.51 | 0.42 |
|  | S3→S5 | 0.220 | -0.27 | 0.62 |
|  | S4→S5 | -0.075 | -0.52 | 0.41 |
|  | S5→S5 | -0.183 | -0.60 | 0.31 |

***Note.*** Autism-related instruments were not administered in this group. S1-S5 = State 1 – State 5. *ρ =* Spearman correlation coefficient. L/UCI = lower/upper 95% confidence intervals; * *p* < .05. ** *p* < .01.

**Supplementary Table S4.** Brain-behaviour associations for non-autistic Proposers from AA/NA dyads.

|  |  | **Reciprocity** | | | **AQ** | | |
| --- | --- | --- | --- | --- | --- | --- | --- |
|  |  | *ρ* | LCI | UCI | *ρ* | LCI | UCI |
| **Reciprocity** |  | 1.000 | -- | -- | -0.450 | -0.78 | 0.08 |
| **AQ** |  | -0.450 | -0.78 | 0.08 | 1.000 | -- | -- |
| **Coverage** | S1 | 0.365 | -0.18 | 0.74 | -0.186 | -0.63 | 0.36 |
|  | S2 | -0.138 | -0.60 | 0.40 | 0.485 | -0.03 | 0.80 |
|  | S3 | -0.297 | -0.70 | 0.25 | 0.034 | -0.48 | 0.53 |
|  | S4 | -0.085 | -0.57 | 0.44 | -0.240 | -0.67 | 0.31 |
| **Occurrence** | S1 | -0.114 | -0.59 | 0.42 | 0.100 | -0.43 | 0.58 |
|  | S2 | -0.177 | -0.63 | 0.36 | 0.472 | -0.05 | 0.79 |
|  | S3 | -0.125 | -0.60 | 0.41 | 0.077 | -0.45 | 0.56 |
|  | S4 | -0.278 | -0.69 | 0.27 | -0.055 | -0.55 | 0.47 |
| **Lifetime** | S1 | 0.479 | -0.04 | 0.79 | -0.263 | -0.68 | 0.28 |
|  | S2 | 0.115 | -0.42 | 0.59 | 0.320 | -0.23 | 0.71 |
|  | S3 | 0.015 | -0.50 | 0.52 | -0.216 | -0.65 | 0.33 |
|  | S4 | 0.159 | -0.38 | 0.62 | -0.234 | -0.66 | 0.31 |
| **Transitions** | S1→S1 | 0.421 | -0.11 | 0.77 | -0.279 | -0.69 | 0.27 |
|  | S2→S1 | -0.147 | -0.61 | 0.39 | 0.135 | -0.40 | 0.60 |
|  | S3→S1 | -0.283 | -0.69 | 0.26 | 0.206 | -0.34 | 0.65 |
|  | S4→S1 | 0.401 | -0.13 | 0.76 | -0.374 | -0.74 | 0.17 |
|  | S1→S2 | 0.312 | -0.23 | 0.71 | 0.355 | -0.19 | 0.73 |
|  | S2→S2 | 0.029 | -0.49 | 0.53 | 0.388 | -0.15 | 0.75 |
|  | S3→S2 | -0.208 | -0.65 | 0.34 | 0.288 | -0.26 | 0.69 |
|  | S4→S2 | -0.223 | -0.66 | 0.32 | 0.077 | -0.45 | 0.56 |
|  | S1→S3 | 0.109 | -0.42 | 0.58 | 0.050 | -0.47 | 0.54 |
|  | S2→S3 | -0.144 | -0.61 | 0.39 | 0.277 | -0.27 | 0.69 |
|  | S3→S3 | -0.074 | -0.56 | 0.45 | -0.125 | -0.60 | 0.41 |
|  | S4→S3 | -0.265 | -0.68 | 0.28 | -0.080 | -0.57 | 0.45 |
|  | S1→S4 | -0.416 | -0.76 | 0.12 | -0.252 | -0.67 | 0.29 |
|  | S2→S4 | 0.164 | -0.38 | 0.62 | 0.213 | -0.33 | 0.65 |
|  | S3→S4 | -0.040 | -0.54 | 0.48 | -0.206 | -0.65 | 0.34 |
|  | S4→S4 | 0.015 | -0.50 | 0.52 | -0.266 | -0.68 | 0.28 |

***Note.*** CARS 2 was not administered in this group. AQ = Autism Spectrum Quotient. S1-S4 = State 1 – State 4; *ρ* = Spearman correlation coefficient. L/UCI = Lower/Upper 95% confidence intervals. * *p* < .05. ** *p* < .01.

**Supplementary Table S5.** Brain-behaviour associations for non-autistic Proposers from NA/NA dyads.

|  |  | **Reciprocity** | | |
| --- | --- | --- | --- | --- |
|  |  | *ρ* | LCI | UCI |
| **Reciprocity** |  | 1.000 | -- | -- |
| **Coverage** | S1 | 0.011 | -0.46 | 0.48 |
|  | S2 | -0.012 | -0.48 | 0.46 |
|  | S3 | **0.521*** | **0.07** | **0.79** |
|  | S4 | -0.343 | -0.70 | 0.15 |
| **Occurrence** | S1 | -0.061 | -0.51 | 0.42 |
|  | S2 | -0.042 | -0.50 | 0.43 |
|  | S3 | **0.561*** | **0.13** | **0.81** |
|  | S4 | -0.036 | -0.49 | 0.44 |
| **Lifetime** | S1 | 0.091 | -0.39 | 0.53 |
|  | S2 | -0.016 | -0.48 | 0.45 |
|  | S3 | 0.214 | -0.28 | 0.62 |
|  | S4 | -0.360 | -0.71 | 0.13 |
| **Transitions** | S1→S1 | -0.011 | -0.48 | 0.46 |
|  | S2→S1 | -0.365 | -0.71 | 0.12 |
|  | S3→S1 | 0.277 | -0.22 | 0.66 |
|  | S4→S1 | 0.136 | -0.35 | 0.57 |
|  | S1→S2 | -0.106 | -0.55 | 0.38 |
|  | S2→S2 | -0.008 | -0.47 | 0.46 |
|  | S3→S2 | 0.188 | -0.31 | 0.60 |
|  | S4→S2 | -0.209 | -0.61 | 0.29 |
|  | S1→S3 | 0.305 | -0.19 | 0.68 |
|  | S2→S3 | 0.313 | -0.18 | 0.68 |
|  | S3→S3 | 0.414 | -0.06 | 0.74 |
|  | S4→S3 | 0.065 | -0.41 | 0.52 |
|  | S1→S4 | -0.141 | -0.57 | 0.35 |
|  | S2→S4 | 0.054 | -0.42 | 0.51 |
|  | S3→S4 | 0.341 | -0.15 | 0.70 |
|  | S4→S4 | -0.403 | -0.73 | 0.08 |

***Note.*** Autism-related instruments were not administered to this group. S1-S4 = State 1 – State 4. *ρ* = Spearman correlation coefficient. L/UCI = Lower/Upper = 95% confidence intervals. * *p* < .05. ** *p* < .01.

**Supplementary Figure S2.** Proportion of variability explained by global signal.


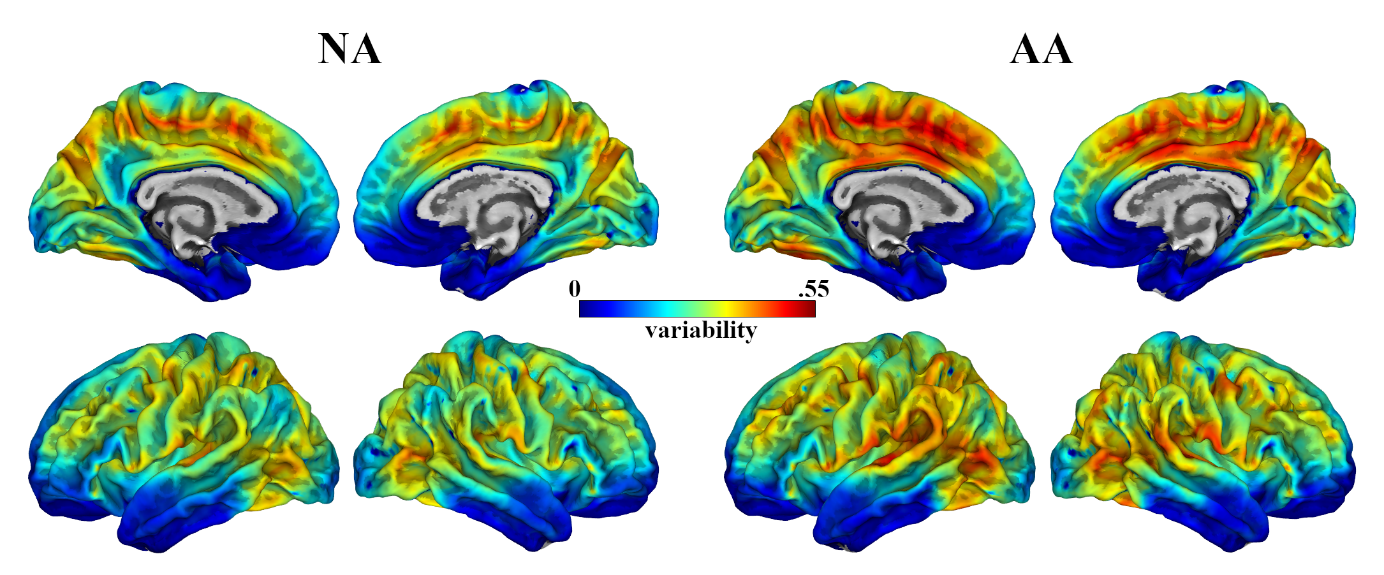


***Note.*** For each participant, the Global signal (GS) was fitted to the time-series extracted from each of the 400 parcels. This determined the proportion of variability shared by GS (the amount of covariance explained by GS) separately for non-autistic (NA; n = 54; *left*) and autistic (AA; n = 16; *right*) adults.

**Supplementary Figure S3.** Similarity between covariance patterns for global signal and latent brain states.

**
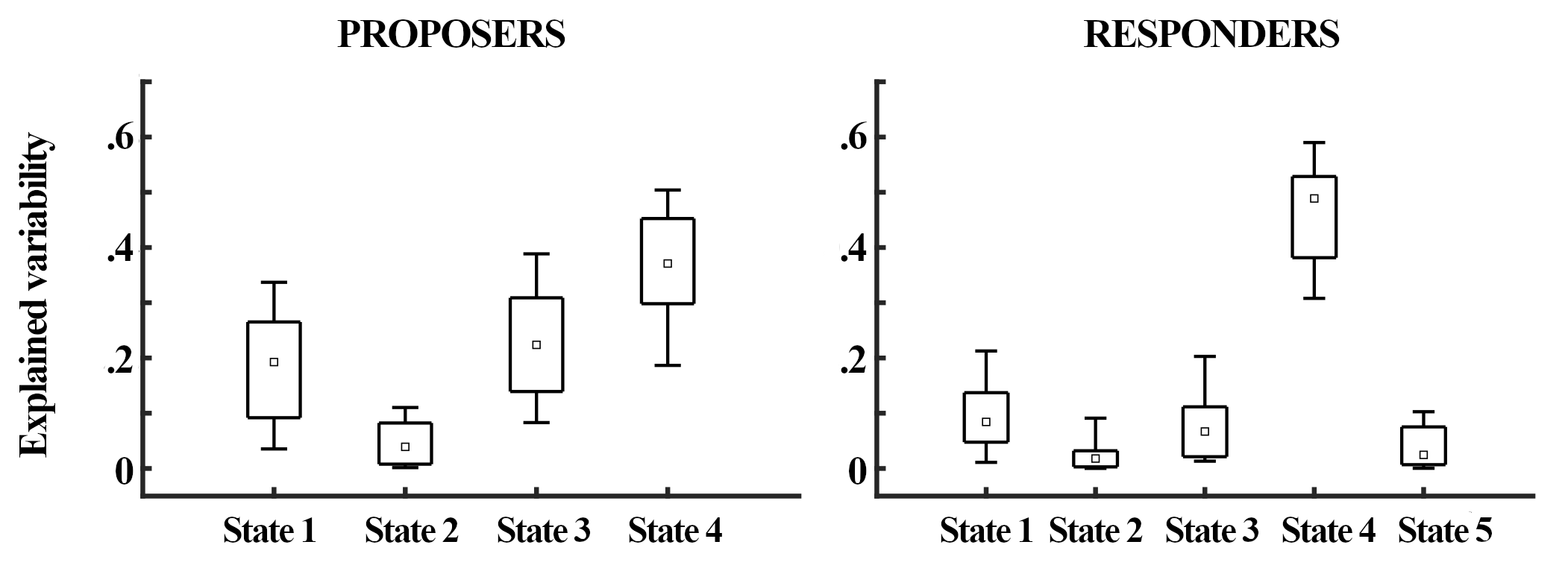
**

***Note****.* Boxplots depict proportion of explained variability for each latent brain state across all participants in each role.

**References**

Beckmann, C. F. (2012). Modelling with independent components. *NeuroImage*, *62*(2), 891–901. https://doi.org/10.1016/j.neuroimage.2012.02.020

Bhaganagarapu, K., Jackson, G. D., & Abbott, D. F. (2013). An automated method for identifying artifact in independent component analysis of resting-state fMRI. *Frontiers in Human Neuroscience*, *7*(JUL), 1–18. https://doi.org/10.3389/fnhum.2013.00343

Jenkinson, M., Beckmann, C. F., Behrens, T. E. J., Woolrich, M. W., & Smith, S. M. (2012). Fsl. *NeuroImage*, *62*(2), 782–790. https://doi.org/10.1016/j.neuroimage.2011.09.015

Patel, A. X., Kundu, P., Rubinov, M., Jones, P. S., Vértes, P. E., Ersche, K. D., Suckling, J., & Bullmore, E. T. (2014). A wavelet method for modeling and despiking motion artifacts from resting-state fMRI time series. *NeuroImage*, *95*, 287–304. https://doi.org/10.1016/j.neuroimage.2014.03.012
